# Food insecurity, epigenetic age acceleration, and depression in middle-aged and older adults: a longitudinal cohort study

**DOI:** 10.3389/fnut.2026.1800352

**Published:** 2026-06-10

**Authors:** Junhua Zhu, Yu Zhang, Jialin Xu, Mengyao Bi, Yikai Hu, Zhengyan Zhang, Zhen Zhou, Zhi Jin

**Affiliations:** 1 The First Affiliated Hospital of Zhengzhou University, Zhengzhou, Henan, China; 2 Center for Clinical Big Data and Analytics Second Affiliated Hospital, Department of Big Data in Health Science School of Public Health, Zhejiang Key Laboratory of Intelligent Preventive Medicine, Zhejiang University School of Medicine, Hangzhou, Zhejiang, China; 3 Department of Hepatobiliary Pancreatic Surgery, Henan Cancer Hospital, Zhengzhou, Henan, China; 4 School of Stomatology, Zhengzhou University, Zhengzhou, Henan, China; 5 College of Public Health, Zhengzhou University, Zhengzhou, Henan, China; 6 School of Public Health and Preventive Medicine, Monash University, Melbourne, VIC, Australia; 7 Department of Neurology, Shanghai Fifth People's Hospital, Fudan University, Shanghai, China; 8 Center of Community-Based Health Research, Fudan University, Shanghai, China

**Keywords:** depression, DNA methylation clocks, epigenetic age acceleration, food insecurity, longitudinal cohort

## Abstract

**Background:**

Depression in middle-aged and older adults is a major contributor to disability, yet upstream social adversity remains underrecognized in prevention. We assessed whether food insecurity (FI) was associated with incident depression and depressive-symptom burden in the US Health and Retirement Study, and whether epigenetic age acceleration (EAA) mediated this association.

**Methods:**

FI was measured using the six-item US Department of Agriculture Food Security Survey Module. Depressive symptoms were assessed with the eight-item Center for Epidemiologic Studies Depression Scale (CES-D-8), with incident depression defined as elevated depressive symptoms using the ≥3 threshold. Associations between FI and incident depression were estimated using multivariable Cox models, and symptom trajectories were analyzed using mixed-effects models. In an epigenetic subsample (*n* = 1,430), 13 DNA methylation clocks were profiled. EAA was derived as age-adjusted residuals and its mediating role was assessed.

**Results:**

Among 5,547 participants included, 1,320 developed incident depression over 7.9 years. Compared with high food security, low and very low food security were associated with higher hazards [hazard ratio (HR) 1.58, 95% confidence interval (CI) 1.32–1.88; and HR 1.94, 95% CI 1.58–2.37], with evidence of a non-linear dose–response and an estimated 9% of incident cases attributable to FI. FI was also associated with a higher depressive-symptom burden (β = 0.72 and 1.59; both *P* < 0.001). Zhang AgeAccel accounted for a small proportion of this association, explaining 5.3% of the total effect.

**Conclusions:**

Food insecurity was associated with higher depression risk and sustained depressive-symptom burden in middle-aged and older adults. EAA accounted for a small proportion of this association.

## 1 Introduction

Depression in middle-aged and older adults is a major contributor to disability and loss of independence, undermining healthy aging (1, 2). The Global Burden of Disease Study 2021 estimated that mental disorders account for 17% of global years lived with disability (YLDs) and 5% of disability-adjusted life years (DALYs), making them the second leading contributor to YLDs worldwide (3). Despite therapeutic advances, prevention has lagged, partly because upstream social determinants of mental health are not routinely measured or incorporated into clinical care and public health surveillance (4, 5). Food insecurity (FI) is one such policy-relevant exposure, reflecting material hardship and structural disadvantage (4, 6). It may affect mental health through sustained psychosocial stress and reduced capacity to maintain health-promoting routines (4, 7, 8). However, the longer-term consequences of FI for depression in middle-aged and older adults remain insufficiently characterized. The biological pathways through which this risk may become embedded are also unclear.

A growing literature links FI to depressive symptoms and depressive disorders across settings (9–11). Meta-analytic evidence suggests that FI is associated with higher odds of depression overall [odds ratio (OR) 1.40, 95% confidence interval (CI) 1.30–1.58], with stronger associations for severe FI (OR 1.77, 95% CI 1.59–1.98) (9). Recent research among adults in Hawaii identified food insecurity as an important socioeconomic factor associated with poor mental health, including depressive symptoms and suicidal ideation (6). Thus, the FI–depression association is increasingly well-established. However, the evidence base is dominated by cross-sectional designs and short follow-up, limiting inference about temporality and within-person change. Depression is also often operationalised as a dichotomous outcome based on a single time-point symptom score, rather than modeled as trajectories that capture persistence, accumulation, or remission over time. Moreover, heterogeneity in risk across sociodemographic and clinical subgroups (e.g., sex, race/ethnicity, and socioeconomic position) remains insufficiently characterized.

Material hardship may become biologically embedded through chronic stress signaling, systemic inflammation, and metabolic dysregulation (12, 13). These processes may contribute to both depression vulnerability and accelerated aging. Epigenetic age acceleration (EAA) may provide a molecular readout of cumulative physiological wear related to the biological embedding of social adversity (14, 15). Consistent with this framework, recent multiethnic evidence suggests that socioeconomic status and lifestyle factors are associated with DNA methylation age, supporting epigenetic aging as a potential marker of social disadvantage (16). Although emerging studies have suggested clock-specific associations between FI and EAA (17, 18), it remains unclear whether EAA prospectively predicts depression or mediates the pathway linking FI to depression in middle-aged and older adults.

To address these gaps, we used longitudinal data from the Health and Retirement Study (HRS). We examined the prospective association between baseline FI and incident depression among middle-aged and older adults. We also evaluated depressive-symptom trajectories and prespecified heterogeneity across population subgroups. In an epigenetic subsample, we profiled multiple EAA measures and evaluated EAA as a potential mediator of the FI–depression association.

## 2 Methods

### 2.1 Study population

The Health and Retirement Study (HRS) is a nationally representative longitudinal cohort of midlife and older adults in the United States, with biennial follow-up (19). In this analysis, baseline food insecurity was assessed in 2013 through the Health Care and Nutrition Study (HCNS), and participants were followed for incident depression across HRS Waves 13–16 (2016–2022). Covariates were drawn from HRS Wave 12 (2014), the closest core HRS interview available around the 2013 HCNS assessment. All participants provided written informed consent, and study procedures were approved by the relevant institutional review board.

We included participants aged ≥45 years with a completed US Department of Agriculture (USDA) six-item food insecurity assessment and follow-up data available to ascertain incident depression. Among 8,073 respondents in the 2013 Health Care and Nutrition Study, we excluded participants aged <45 years or with missing age information (*n* = 116), those with missing food insecurity data (*n* = 494), those with prevalent depression at baseline (*n* = 1,417), and those with missing depression outcome data during follow-up (*n* = 499), yielding a primary analytic sample of 5,547 participants (Supplementary Figure 1). For analyses incorporating epigenetic aging, we further restricted to participants with available genome-wide DNA methylation–derived measures and without incident depression occurring at HRS Wave 13 (*n* = 1,430).

### 2.2 Food insecurity

Household food insecurity was assessed at baseline (2013) using the USDA six-item short form of the Household Food Security Survey Module (12-month recall), administered in HCNS (20). The instrument captures a graded spectrum of food-related hardship, including food not lasting and inability to afford balanced meals, economically driven meal cutting or skipping (with frequency), reduced intake, and hunger without eating. Items were scored according to USDA guidance (affirmative = 1; non-affirmative = 0) and summed to generate a severity score (range 0–6), with higher scores indicating greater food insecurity. Consistent with established cut-points (20, 21), participants were classified as having high food security (0–1), low food security (2–4), or very low food security (5–6). Item wording and scoring are provided in Supplementary Table 1.

### 2.3 Depressive symptoms and incident depression

Depressive symptoms were assessed biennially from HRS Wave 12 through Wave 16 using the eight-item Center for Epidemiologic Studies Depression scale (CES-D-8) (22, 23), which captures whether each symptom was experienced much of the time during the week prior to interview. The CES-D-8 is a brief version of the original 20-item CES-D and has been widely used in HRS-based studies and other large aging cohorts as an efficient measure of depressive symptoms (24–26). The CES-D-8 includes six negative-affect/somatic symptoms (felt depressed, everything was an effort, restless sleep, could not get going, felt lonely, felt sad) and two positive-affect items (was happy, enjoyed life). Items were scored yes = 1 and no = 0, with the two positive-affect items reverse-coded, and summed to yield a total score ranging from 0 to 8 (higher scores indicate greater symptom burden). For the primary time-to-event analyses, depression was defined as elevated depressive symptoms (CES-D-8 ≥3), and incident depression was ascertained as the first follow-up wave meeting this threshold among participants below it at baseline (27, 28). This threshold was used to indicate elevated depressive symptoms consistent with probable depression rather than a formal clinical diagnosis (26, 29). To evaluate changes in symptom burden over follow-up by baseline food security status, we analyzed repeated CES-D-8 scores using linear mixed-effects models including food security status, time, and their interaction, adjusted for the same covariates as in the primary models. Item wording and scoring are provided in Supplementary Table 2.

### 2.4 Epigenetic clocks and epigenetic age acceleration

DNA methylation measures from the HRS 2016 Venous Blood Study were used to derive epigenetic aging indices based on the Infinium MethylationEPIC platform, following the HRS epigenetic clocks protocol (30–32). Further details on blood collection and processing are available online through HRS (33). We considered thirteen published DNAm clocks spanning (i) first-generation predictors trained on chronological age (34–42), (ii) phenotype- or mortality-informed clocks (43–45), and (iii) a methylation-based pace-of-aging measure (46) (Supplementary Table 3). Because these measures are reported on different native scales (years, scores, or rate), we retained each clock in its original metric and used harmonized transformations for regression analyses as specified below.

For clocks expressed in years, epigenetic age acceleration (EAA) was defined as the standardized residual from regressing DNAm age on chronological age at the 2016 assessment, such that positive values indicate accelerated epigenetic aging relative to chronological age (47, 48). DunedinPoAm was analyzed as a rate measure (years of physiological decline per chronological year) (46), where higher values indicate faster aging. Definitions and measurement characteristics of all clocks are provided in Supplementary Table 3.

### 2.5 Covariates

Covariates were prespecified as potential confounders of the food insecurity–incident depression association, spanning demographic, socioeconomic, and lifestyle factors. Unless otherwise stated, covariates were obtained from the HRS core interview at Wave 12 and included age at baseline (years), sex, race/ethnicity (White, Black, Hispanic, other), education (below high school, high school, college or above), marital status (married or partnered; separated/divorced/widowed; never married), smoking status (never vs ever; ever smokers included former and current smokers), drinking status (never vs ever), physical activity (vigorous, moderate, inactive), residence (urban, suburban, rural), household income (low, moderate, high; grouped in tertiles), and body mass index (BMI; kg/m^2^). Physical activity categories were defined using self-reported frequency: vigorous activity more than once per week (vigorous), moderate activity more than once per week (moderate), and all others classified as inactive. Histories of major chronic diseases (hypertension, diabetes, heart disease, stroke, and cancer) were summarized at baseline and evaluated in sensitivity analyses rather than adjusted in the main analyses.

### 2.6 Statistical analysis

Baseline characteristics are presented as means (SDs) for continuous variables and *n* (%) for categorical variables. Missing covariate data were imputed using multiple imputation by chained equations (MICE; *m* = 5); the imputation model included food insecurity, depression indicators, and all covariates, and estimates were pooled using Rubin's rules (49).

Multivariable Cox proportional hazards models estimated hazard ratios (HRs) and 95% confidence intervals (CIs) for baseline FI, using time since baseline (years) to incident depression, death, loss to follow-up, or end of follow-up; ties were handled using the Efron method. FI was modeled categorically (high food security [reference], low, very low) and continuously (per one-point increase), with trend tests treating categories as an ordinal term. The proportional hazards assumption was assessed using Schoenfeld residuals (50). The non-linearity of association was evaluated using restricted cubic splines with three knots placed at prespecified percentiles, and cumulative incidence was visualized using Kaplan–Meier curves, with between-group differences assessed by two-sided log-rank tests. Subgroup analyses were performed by major depression risk factors.

We examined Spearman correlations among the six USDA items (Supplementary Figure 2), evaluated each component separately and jointly in mutually adjusted models, and tested effect modification in subgroups using multiplicative interaction terms. Robustness was assessed in prespecified sensitivity analyses, including complete-case analyses, additional adjustment for major chronic diseases, exclusion of participants with baseline major chronic diseases, exclusion of events within the first 2 years, a 5-year landmark analysis conditioned on being event-free at 5 years, and propensity score–matched analyses with assessment of overlap (51) (Supplementary Figure 3). E-values were computed for point estimates and lower confidence bounds (52). Repeated CES-D-8 scores (Wave 12–16) were analyzed using linear mixed-effects models with participant-specific random intercepts, including fixed effects for food security status, time, and their interaction.

In the epigenetic subsample, we analyzed 13 DNA methylation–derived aging measures (Supplementary Table 3). Clock outputs were retained in their native metrics; residual-based epigenetic age acceleration (EAA) was defined as residuals from regressing each DNAm-based measure on chronological age at the 2016 DNAm assessment and was standardized (per one SD) for association analyses, whereas DunedinPoAm was analyzed on its native pace scale. Model assumptions were checked using residual–fitted and Q–Q plots (53), Durbin–Watson tests for independence (54), and variance inflation factors for multicollinearity (Supplementary Figures 4–6). Associations of FI with epigenetic measures were estimated using multivariable linear regression, and associations of epigenetic measures with incident depression were estimated using Cox models. Mediation analyses decomposed total effects into natural direct and indirect effects through epigenetic measures showing evidence of association with both baseline FI and incident depression (55), with uncertainty quantified by bootstrap resampling (1,000 iterations). All 13 epigenetic aging measures were evaluated before selecting the mediation candidate.

All statistical tests were two-sided, with *P* < 0.05 considered statistically significant. Analyses were performed using R software (version 4.5.1; R Foundation for Statistical Computing, Vienna, Austria).

## 3 Results

### 3.1 Participant characteristics at baseline

This study included 5,547 participants free of depression at baseline, who were followed up for a median of 7.9 years [interquartile range (IQR), 7.6–8.2]. At baseline, 4,789 participants (86.3%) had high food security, whereas 468 (8.4%) and 290 (5.2%) had low and very low food security, respectively. The mean age was 66.5 years [standard deviation (SD) 10.3], 57.8% were women, and mean body mass index (BMI) was 28.5 kg/m^2^ (SD 5.8). During follow-up, 1,320 participants (23.8%) developed incident depression, increasing stepwise with worsening food security (21.4%, 35.7%, and 43.5% for high, low, and very low food security; Table 1; Supplementary Table 4).

**Table 1:** Baseline characteristics of participants by food security status.

Variables	Total (*n* = 5,547)	High food security (*n* = 4,789)	Low food security (*n* = 468)	Very low food security (*n* = 290)	*P*-value
Age at baseline, years	66.51 ± 10.28	67.26 ± 10.25	62.75 ± 9.69	60.33 ± 8.06	**<0.001**
BMI, kg/m^2^	28.47 ± 5.83	28.21 ± 5.70	29.99 ± 5.91	30.26 ± 6.98	**<0.001**
Sex			
Male	2,341 (42.20)	2,055 (42.91)	177 (37.82)	109 (37.59)	**0.03**
Female	3,206 (57.80)	2,734 (57.09)	291 (62.18)	181 (62.41)	
Race			
White	3,981 (71.77)	3,668 (76.59)	191 (40.81)	122 (42.07)	**<0.001**
Black	856 (15.43)	623 (13.01)	151 (32.26)	82 (28.28)	
Hispanic	526 ( 9.48)	355 ( 7.41)	102 (21.79)	69 (23.79)	
Other	184 ( 3.32)	143 ( 2.99)	24 ( 5.13)	17 ( 5.86)	
Education			
Below high school	740 (13.34)	520 (10.86)	137 (29.27)	83 (28.62)	**<0.001**
High school	1,792 (32.31)	1,533 (32.01)	165 (35.26)	94 (32.41)	
College or above	3,015 (54.35)	2,736 (57.13)	166 (35.47)	113 (38.97)	
Marital status			
Married or partnered	3,775 (68.05)	3,347 (69.89)	272 (58.12)	156 (53.79)	**<0.001**
Separated/divorced/widowed	1,558 (28.09)	1,288 (26.89)	163 (34.83)	107 (36.90)	
Never married	214 ( 3.86)	154 ( 3.22)	33 ( 7.05)	27 ( 9.31)	
Smoking status			
Never smokers	2,605 (46.96)	2,289 (47.80)	207 (44.23)	109 (37.59)	**<0.01**
Ever smokers	2,942 (53.04)	2,500 (52.20)	261 (55.77)	181 (62.41)	
Drinking status			
Never drinkers	2,376 (42.83)	1,952 (40.76)	275 (58.76)	149 (51.38)	**<0.001**
Ever drinkers	3,171 (57.17)	2,837 (59.24)	193 (41.24)	141 (48.62)	
Physical activity			
Inactive	1,455 (26.23)	1,228 (25.64)	134 (28.63)	93 (32.07)	**<0.01**
Moderate	1,866 (33.64)	1,600 (33.41)	180 (38.46)	86 (29.66)	
Vigorous	2,226 (40.13)	1,961 (40.95)	154 (32.91)	111 (38.28)	
Residence			
Urban	2,869 (51.72)	2,464 (51.45)	237 (50.64)	168 (57.93)	0.18
Suburban	1,207 (21.76)	1,049 (21.90)	109 (23.29)	49 (16.90)	
Rural	1,471 (26.52)	1,276 (26.64)	122 (26.07)	73 (25.17)	
Household income			
Low	1,849 (33.33)	1,358 (28.36)	290 (61.97)	201 (69.31)	**<0.001**
Moderate	1,847 (33.30)	1,639 (34.22)	137 (29.27)	71 (24.48)	
High	1,851 (33.37)	1,792 (37.42)	41 ( 8.76)	18 ( 6.21)	
Hypertension	3,206 (57.80)	2,732 (57.05)	308 (65.81)	166 (57.24)	**<0.01**
Diabetes	1,176 (21.20)	969 (20.23)	142 (30.34)	65 (22.41)	**<0.001**
Heart disease	1,234 (22.25)	1,075 (22.45)	96 (20.51)	63 (21.72)	0.62
Stroke	401 ( 7.23)	331 ( 6.91)	42 ( 8.97)	28 ( 9.66)	0.07
Cancer	880 (15.86)	797 (16.64)	51 (10.90)	32 (11.03)	**<0.001**
Incident depression during follow-up	1,320 (23.80)	1,027 (21.44)	167 (35.68)	126 (43.45)	**<0.001**

Continuous variables are presented as mean ± standard deviation (SD), and categorical variables are expressed as number [percentage (%)]. The proportion of missing data was as follows: body mass index (2.02%), smoking status (1.42%), cancer (1.24%), physical activity level (1.17%), residence (1.06%), marital status (1.05%), Drinking status (1.03%), household income (0.99%), hypertension (0.99%), diabetes (0.99%), heart disease (0.99%), and stroke (0.99%). All other variables included in this table were complete with no missing values.

Two-sided P values are presented without adjustment for multiple comparisons, with P values below 0.001 reported as <0.001. The bold values indicate statistically significant P-values (P < 0.05).

Compared with those with high food security, participants with low or very low food security were more likely to be from racial/ethnic minority groups and to have lower educational attainment and household income, and were, on average, younger. They also showed a less favorable behavioral and clinical profile, including higher BMI, more ever smoking, lower vigorous physical activity, and a higher prevalence of hypertension and diabetes, whereas residence differed little across categories (Table 1). Baseline characteristics and depression incidence in the epigenetic subsample (*n* = 1,430) were broadly similar to those in the full analytic sample (Supplementary Table 5).

### 3.2 Food insecurity and risk of incident depression

**Figure 1:**
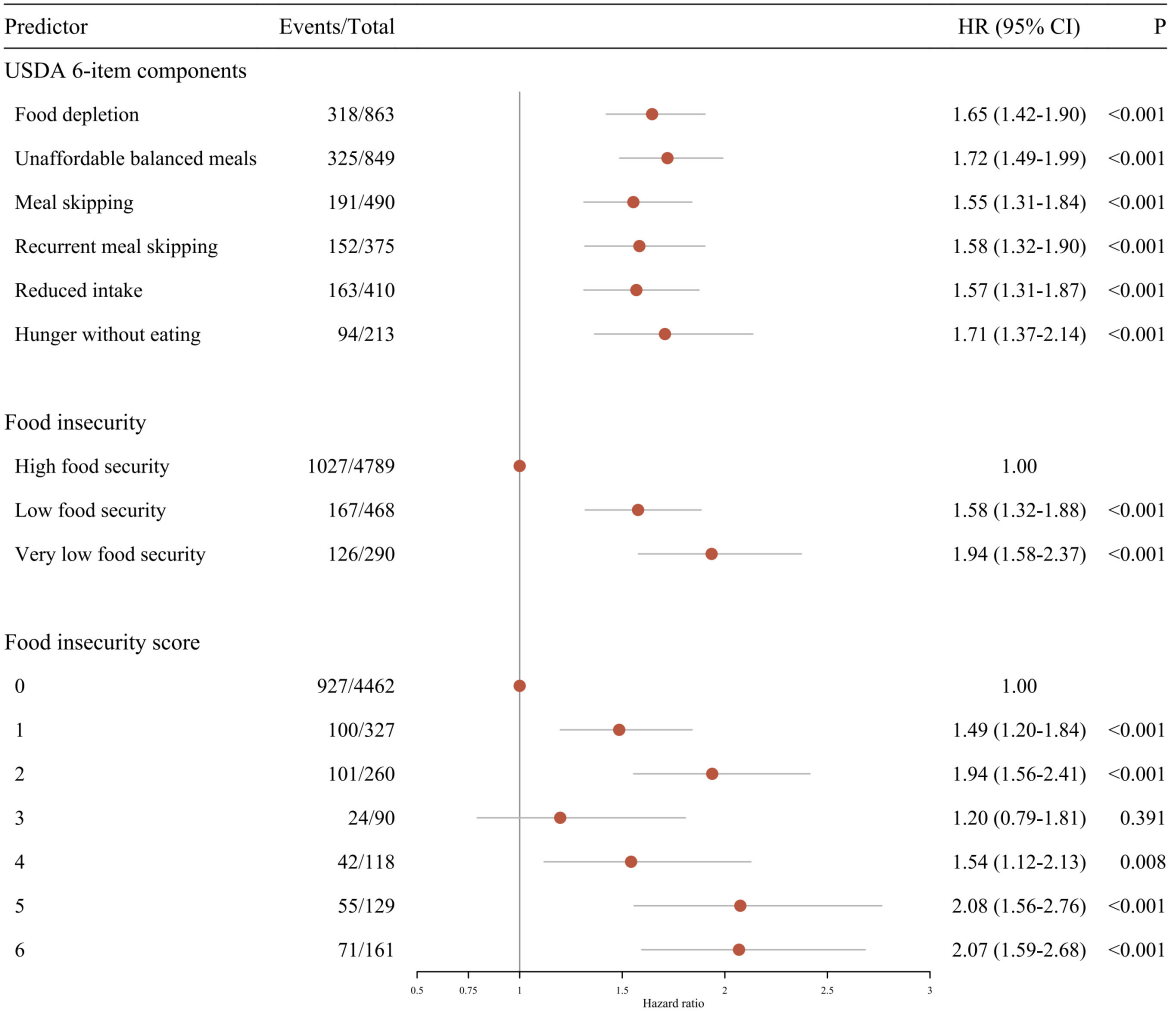
Association between food insecurity and risk of incident depression. Data are from 5,547 participants in the Health and Retirement Study with complete baseline assessments of food security. Food insecurity was measured using the USDA six-item scale and categorized as high (score 0–1), low (score 2–4), or very low (score 5–6) food security. The upper panel shows associations between individual USDA food insecurity components and incident depression. The middle panel displays associations for categorical food security status, with high food security as the reference group. The lower panel illustrates associations across levels of the food insecurity score. Hazard ratios (HRs) and 95% confidence intervals (CIs) were estimated using multivariable Cox proportional hazards models adjusted for age at baseline, sex, race, education, marital status, smoking status, drinking status, physical activity, residence, household income, body mass index, hypertension, diabetes, heart disease, stroke, and cancer. Points indicate HRs and horizontal bars represent 95% CIs. Two-sided unadjusted *P* values are shown.

The analysis using fully-adjusted Cox model showed that, compared with high food security, low and very low food security were associated with 58% and 94% higher risk of depression, respectively [hazard ratio (HR) 1.58, 95% confidence interval (CI) 1.32–1.88; and HR 1.94, 1.58–2.37; Figure 1], after multivariable adjustment for prespecified demographic, socioeconomic and lifestyle factors. Kaplan–Meier curves separated early and remained ordered across food security categories throughout follow-up (log-rank *P* < 0.001; Supplementary Figure 7). When the food insecurity score was modeled continuously using restricted cubic splines, a non-linear dose–response association was observed (*P*_overall_ <0.0001; *P*_non − linear_ = 0.006). The risk increased steeply at USDA scores of 1–4 when compared a score of zero, then plateaued at scores above 4 (Supplementary Figure 8).

All six USDA items were associated with incident depression individually (Figure 1; Supplementary Table 7), consistent with moderate-to-strong inter-item correlations (Supplementary Figure 2). In mutually adjusted models including all six items simultaneously, food depletion and unaffordable balanced meals retained independent associations with incident depression (HR 1.25, 95% CI 1.01–1.56; and 1.43, 1.16–1.77; Supplementary Table 7), whereas the association with remaining items were attenuated. Under model assumptions, population-attributable fraction estimates suggested that food insecurity accounted for ~9% of incident depression cases (Supplementary Table 7). E-values for the categorical associations were 2.54 (low food security) and 3.29 (very low food security), with corresponding lower confidence-bound E-values of 1.97 and 2.54, respectively (Supplementary Table 8).

### 3.3 Subgroup and sensitivity analyses

**Figure 2:**
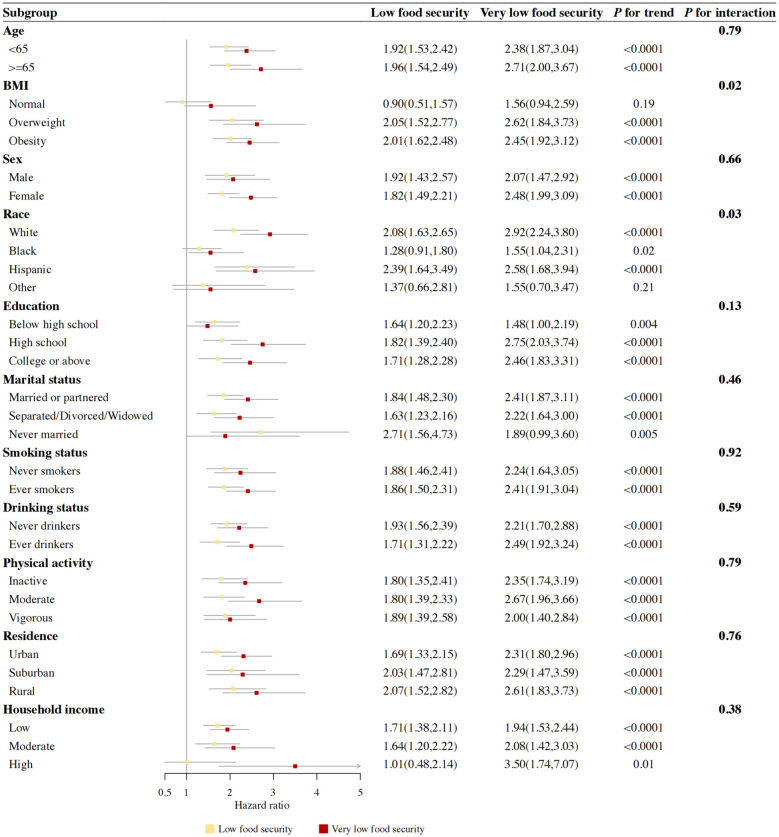
Associations between food insecurity and incident depression across population subgroups. Hazard ratios (HRs) and 95% confidence intervals (CIs) for incident depression are shown for low food security and very low food security compared with high food security (reference) across prespecified subgroups of age group, body mass index group, sex, race, education, marital status, smoking status, drinking status, physical activity, residence, and household income. Estimates were derived from multivariable Cox proportional hazards models adjusted for age group, body mass index group, sex, race, education, marital status, smoking status, drinking status, physical activity, residence, and household income, except for the stratifying variable in each subgroup analysis. Age group and body mass index group were entered as categorical variables. *P* for trend was calculated by modeling food security status as an ordinal variable. *P* for interaction was obtained from models including a multiplicative interaction term between food security status and the subgroup variable. Two-sided unadjusted *P* values are shown, with values <0.001 reported as <0.001.

Food insecurity was consistently associated with a higher risk of incident depression in most of prespecified subgroups (Figure 2; Supplementary Figure 9). Evidence of effect modification was detected for body mass index (BMI; *P*_interaction_ = 0.02) and race/ethnicity (*P*_interaction_ = 0.03), whereas interactions with age, sex, education, marital status, smoking and drinking status, physical activity, residence, and household income were not supported (all *P*_interaction_ > 0.05).

Subgroup patterns suggested effect modification by BMI and race/ethnicity, with otherwise broadly consistent associations across strata. Associations were minimal among normal-weight participants [low food security HR 0.90 (95% CI 0.51–1.57); very low HR 1.56 (0.94–2.59)] but stronger among those overweight or living with obesity, with HRs exceeding two for very low food security [overweight 2.62 (1.84–3.73); obesity 2.45 (1.92–3.12)]. By race/ethnicity, estimates were most pronounced among White and Hispanic participants [very low HR 2.92 (2.24–3.80) and 2.58 (1.68–3.94), respectively] and attenuated among Black participants [1.55 (1.04–2.31)], with similar patterns when modeling food insecurity continuously (Supplementary Figure 9).

Sensitivity analyses yielded similar results to the main analysis across six prespecified scenarios (Supplementary Table 12), with HRs typically spanning 1.46–1.62 for low and 1.88–1.99 for very low food security. Notably, associations strengthened after excluding participants with baseline major chronic diseases (low HR 2.11; very low HR 2.36). Continuous-score analyses were similarly stable (HR_per1 − point_ ≈ 1.13–1.18; Supplementary Table 12).

### 3.4 Food insecurity and longitudinal trajectories of depressive symptoms

**Figure 3:**
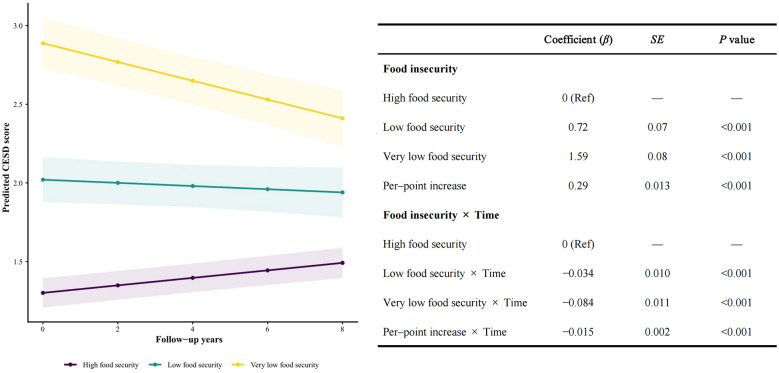
Trajectories of depressive symptoms from Wave 12 to Wave 16 by baseline food security status. Predicted CES-D scores across follow-up (Wave 12–16; 0–8 years) are shown for participants categorized at baseline using the USDA six-item scale as high food security, low food security, or very low food security. Lines denote model-based predicted mean CES-D scores and shaded bands indicate 95% confidence intervals. Regression coefficients (β), standard errors (*SE*) and two-sided *P* values are from linear mixed-effects models including main effects for food insecurity, time, and their interaction (food insecurity × time), adjusted for age at baseline, sex, race, education, marital status, smoking status, drinking status, physical activity, residence, household income, and body mass index. *P* values <0.001 are reported as <0.001.

In longitudinal analyses of repeated CES-D-8 assessments (Waves 12–16), baseline food insecurity was associated with higher depressive symptom burden in a graded manner (Figure 3). In covariate-adjusted mixed-effects models, CES-D-8 scores were higher among participants with low and very low food security than among those with high food security (β = 0.72 and 1.59; both *P* < 0.001), with consistent results when modeling food insecurity continuously (per one-point increase, β = 0.29; *P* < 0.001).

Trajectories over follow-up differed modestly by baseline food security status (Figure 3). Time-interaction terms indicated differential symptom change, with more negative slopes in the low and very low food security groups relative to the high–food security reference (β for food insecurity × time = −0.034 and −0.084; per-point × time = −0.015; all *P* < 0.001), suggesting a modest narrowing of between-group differences over time while rank ordering was largely preserved.

### 3.5 Epigenetic age acceleration and mediation of the food insecurity–depression association

**Figure 4:**
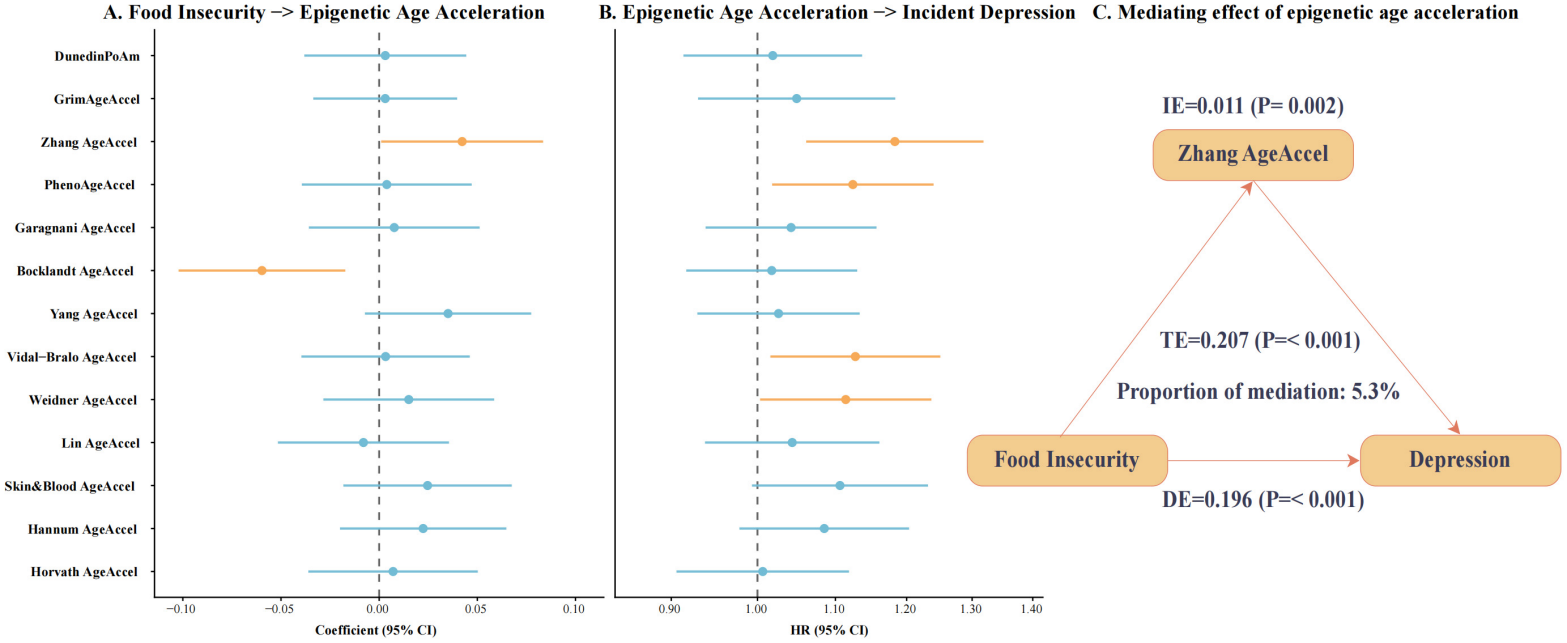
Epigenetic age acceleration as a mediator of the association between food insecurity and incident depression. **(A)** Associations between baseline food insecurity and epigenetic age acceleration measures. Points indicate adjusted regression coefficients (β) and horizontal lines denote 95% confidence intervals (CIs); the dashed vertical line marks the null (β = 0). **(B)** Associations between epigenetic age acceleration measures and incident depression. Points indicate hazard ratios (HRs) and horizontal lines denote 95% CIs; the dashed vertical line marks the null (HR = 1). **(C)** Mediation analysis for the epigenetic clock meeting the criteria of association with both food insecurity and incident depression (Zhang AgeAccel). The indirect effect (IE) represents the pathway through Zhang AgeAccel; the direct effect (DE) represents the association not through Zhang AgeAccel; the total effect (TE) represents the overall food insecurity–depression association; and the proportion mediated represents the percentage of the total effect explained by Zhang AgeAccel. Models in **(A, B)** were adjusted for age at baseline, sex, race, education, marital status, smoking status, drinking status, physical activity, residence, household income, and body mass index. Two-sided unadjusted *P* values are shown, with values <0.001 reported as <0.001.

To assess whether epigenetic age acceleration (EAA) might sit on the pathway linking food insecurity to subsequent depression, we profiled a panel of EAA measures in the epigenetic subsample (Figure 4A; Supplementary Table 9). Food insecurity mapped onto a clock-specific, rather than pervasive, pattern: per one-point higher food insecurity score, Zhang AgeAccel showed modest, nominal evidence of association (β = 0.042, 95% CI 0.001–0.083), whereas most other measures showed little support for an association.

We then tested whether EAA predicted incident depression prospectively (Figure 4B; Supplementary Table 10). Zhang AgeAccel was associated with higher depression risk (HR = 1.18, 95% CI 1.06–1.32 per one-SD), and some second-generation measures also showed associations with incident depression (e.g., PhenoAgeAccel: HR = 1.12, 1.02–1.24). Tertile-based analyses were directionally consistent. However, Zhang AgeAccel was the only measure showing concordant evidence across both the food insecurity → AgeAccel and AgeAccel → depression steps, and was therefore carried forward for mediation analysis in this multi-clock setting.

Mediation analyses under the counterfactual framework supported partial mediation through Zhang AgeAccel (Figure 4C; Supplementary Table 11). The natural indirect effect (NIE) was small but statistically supported (NIE = 1.011, 95% CI 1.003–1.022), corresponding to 5.3% (95% CI 1.5–11.7) of the total effect (TE = 1.230, 1.163–1.304), while the natural direct effect (NDE) remained the dominant component (NDE = 1.217, 1.148–1.292). Collectively, these results suggest that accelerated epigenetic aging—captured here by Zhang AgeAccel—accounts for a limited yet detectable fraction of the excess depression risk associated with food insecurity.

## 4 Discussion

In this longitudinal cohort, baseline food insecurity (FI) was associated with elevated risk of incident depression and higher depressive-symptom burden over follow-up. Associations were stable after adjustment for sociodemographic factors, health behaviors and cardiometabolic profiles, and were consistent across sensitivity analyses intended to mitigate reverse causation. In the epigenetic subsample, epigenetic age acceleration (EAA), most consistently captured by Zhang AgeAccel, showed concordant associations with FI and subsequent depression and supported a small indirect effect, compatible with partial biological embedding under the assumptions required for mediation inference. Together, these findings position FI as a policy-relevant upstream determinant of depression risk and depressive-symptom burden in middle-aged and older adults, with implications for food assistance, social protection, and community-based support for vulnerable aging populations.

Evidence from systematic reviews and multi-country analyses converges on a consistent pattern: adults experiencing FI have higher depression risk, even though much of the literature remains cross-sectional (10, 56–59). For instance, a systematic review and meta-analysis of US studies reported higher odds of depression among food-insecure adults, adjusted odds ratio (OR) 2.05 with 95% confidence interval (CI) 1.73–2.43 (10). Longitudinal evidence has further raised the possibility of bidirectionality: in a US multi-city cohort of mothers, baseline depression predicted subsequent FI (OR 1.53) and baseline FI predicted subsequent depression (OR 1.36) (56). In another nationally representative study across six low- and middle-income countries among adults aged ≥50 years, severe FI was associated with more than doubled odds of Diagnostic and Statistical Manual of Mental Disorders, Fourth Edition (DSM-IV) depression (OR 2.43), whereas moderate FI showed a weaker, non-significant association (OR 1.69) (57). Complementing individual-level measures, geospatial evidence from South Africa indicates that residing in FI “hotspot” communities was associated with higher subsequent depression incidence, adjusted risk ratio (aRR) 1.11 with 95% CI 1.01–1.22, even after accounting for household FI (58). Against this backdrop, our study refines the risk structure in a nationally representative US aging cohort by delineating severity-graded and partially non-linear associations with incident depression, hazard ratio (HR) 1.58 for low and 1.94 for very low vs. high food security, and by showing that FI relates not only to onset but also to sustained depressive-symptom burden over time; EAA provides modest mechanistic coherence consistent with partial biological embedding.

Although FI is often treated as a common exposure, our subgroup analyses suggest potential heterogeneity in its association with incident depression. We observed effect modification by BMI and race/ethnicity, with *P*_interaction_ = 0.02 and 0.03, respectively, and little support for modification across other strata. The BMI-related pattern was particularly notable: relative to high food security, low and very low food security were associated with higher hazards among participants with overweight (HR 2.05 and 2.62) or obesity (HR 2.01 and 2.45), whereas estimates were weaker and less precise among those with normal weight (HR 0.90 and 1.56). This pattern is consistent with the coexistence of FI and excess adiposity in high-income settings (60, 61). Constrained resources may increase reliance on low-cost, energy-dense, nutrient-poor foods and reduce access to higher-quality foods (62, 63). Irregular eating patterns, chronic stress, sleep disruption, and metabolic adaptation may further link FI to both excess adiposity and depression vulnerability (64–66). Thus, elevated BMI may help identify a subgroup in whom material hardship, poorer dietary quality, obesity-related inflammatory–metabolic dysregulation, and depressive risk converge (62, 67). Heterogeneity by race/ethnicity should be interpreted cautiously, because race and ethnicity are social rather than biological categories (68, 69). These patterns may reflect structural and contextual disadvantage, including differences in neighborhood food environments, discrimination, healthcare access, and buffering resources, rather than inherent group differences (70, 71). These subgroup findings should be considered hypothesis-generating and require replication in cohorts with more detailed dietary, metabolic, and contextual measures.

Material hardship may become biologically embedded through sustained activation of stress pathways and downstream inflammatory–metabolic and immune dysregulation, processes plausibly linked to depression risk in middle-aged and older adults (72). Within this framework, EAA can be viewed as an integrative readout of cumulative physiological wear rather than a single downstream biomarker (73–75). The clock-specific pattern we observed is biologically coherent: epigenetic clocks differ in their training targets and therefore in sensitivity to social and environmental exposures, with mortality- or risk-informed clocks plausibly more responsive to material adversity than first-generation chronological-age clocks (76, 77). As a mortality-associated DNA methylation measure, Zhang AgeAccel may capture risk-related aging processes involving stress-related, immune–metabolic, or mortality-linked vulnerability (45). Among the 13 clocks evaluated, Zhang AgeAccel was the only measure showing concordant evidence across both prerequisites for mediation—associations with FI and with subsequent depression—and was therefore carried forward for mediation analysis. The resulting indirect effect was small but statistically supported. Such modest mediation is consistent with partial biological embedding rather than a dominant mechanism, given depression's multi-pathway etiology and the likelihood that FI operates through multiple channels, including psychological stress, behavioral constraints, healthcare access and comorbidity (78, 79). These inferences warrant caution because methylation was assessed at a single time point and mediation rests on strong assumptions. Future work should test temporality using repeated measures of FI, DNA methylation, and depressive symptoms, integrate proximal intermediates such as inflammatory–metabolic and immune-aging markers, and leverage policy changes or natural experiments to probe reversibility of aging signatures and downstream depression risk.

The study is strengthened by its prospective design in a nationally representative aging cohort, standardized assessment of FI, and repeated measures of depressive symptoms across multiple waves, which together allow evaluation of both incident depression and symptom trajectories. The main inferences were reinforced by consistent results across robustness analyses, and the multi-clock DNA methylation panel adds a complementary biological lens for interpreting the observed associations. However, several limitations should be considered. FI was measured at baseline only and may fluctuate with household circumstances and policy environments, so time-varying misclassification could have attenuated effect estimates; studies capturing transitions into and out of FI will be particularly informative. Covariates were obtained from HRS Wave 12, the closest core interview after the 2013 HCNS assessment; therefore, some temporal ambiguity in covariate measurement may remain, although most covariates were relatively stable sociodemographic or health-related characteristics. Depression was defined using the CES-D-8 threshold rather than a clinical diagnosis, which may have introduced outcome misclassification and provided less detailed symptom assessment than the original 20-item CES-D. As with any observational analysis, residual confounding and bidirectional relationships cannot be fully ruled out despite extensive adjustment and efforts to mitigate reverse causation. The epigenetic analyses were confined to a biomarker subsample with single time-point methylation profiling, which limits generalisability and prevents assessment of within-person change in epigenetic aging. Finally, mediation estimates rely on strong assumptions and should be interpreted as supportive evidence consistent with partial biological embedding rather than definitive proof of causality.

## 5 Conclusions

In conclusion, FI was prospectively associated with incident depression and sustained depressive-symptom burden among middle-aged and older US adults. These findings support FI as an upstream and potentially modifiable social determinant of mental health, with implications for food assistance, social protection, and community-based support in vulnerable aging populations. Zhang AgeAccel explained only a small proportion of this association, consistent with partial biological embedding rather than a dominant mechanism. Future studies should evaluate whether interventions and policies that improve food security reduce downstream depression burden and related aging signatures.

## Data Availability

The data analyzed in this study are available from the Health and Retirement Study (HRS; https://hrs.isr.umich.edu/data-products). Public-use HRS files are available to registered users through the HRS data portal, whereas restricted or sensitive data, including DNA methylation and epigenetic measures, require application and approval through HRS. The authors are not permitted to redistribute these data.
