# Supplementary material for: Food insecurity, epigenetic age acceleration, and depression in middle-aged and older adults: a longitudinal cohort study
Source: Front Nutr. 2026 Jun 10;13:1800352. doi: 10.3389/fnut.2026.1800352 (PMC13290441; doi:10.3389/fnut.2026.1800352)
Supplement: Supplementary file 1 [file Table_1.docx]

**Supplementary materials**

**Supplementary Tables**

Supplementary Table 1. USDA six-item food insecurity scale (HCNS 2013): items, wording, and scoring

Supplementary Table 2. Assessment of depressive symptoms and depression: items, wording, and scoring criteria

Supplementary Table 3. Definitions and characteristics of epigenetic aging measures

Supplementary Table 4. Baseline characteristics according to incident depression status

Supplementary Table 5. Baseline characteristics of the full analytic sample and the epigenetic subsample

Supplementary Table 6. Distributions of epigenetic clocks and measures of epigenetic age acceleration

Supplementary Table 7. Associations of food insecurity and its individual components with incident depression

Supplementary Table 8. E-values for the association between food insecurity severity and incident depression

Supplementary Table 9. Associations between food insecurity and epigenetic age acceleration

Supplementary Table 10. Associations between epigenetic age acceleration and incident depression

Supplementary Table 11. Mediation analyses evaluating accelerated epigenetic aging in the association between food insecurity and incident depression

Supplementary Table 12. Sensitivity analyses for the association between food insecurity and incident depression

**Supplementary Figures**

Supplementary Figure 1. Flowchart of participant selection

Supplementary Figure 2. Spearman correlations among items of the USDA six-item food insecurity scale

Supplementary Figure 3. Propensity score distributions across food insecurity

categories to assess the positivity assumption

Supplementary Figure 4. Residual-versus-fitted plots for models of epigenetic aging measures

Supplementary Figure 5. Quantile–quantile plots for models of epigenetic aging measures

Supplementary Figure 6. Durbin–Watson statistics for models of epigenetic aging measures

Supplementary Figure 7. Kaplan–Meier curves for incident depression by baseline food security status

Supplementary Figure 8. Dose–response association between food insecurity score and incident depression

Supplementary Figure 9. Stratified associations between food insecurity score and incident depression across demographic and clinical subgroups

**Supplementary Table 1. USDA six-item food insecurity scale (HCNS 2013): items, wording, and scoring**

| **USDA 6-item component** | **Questionnaire wording** | **Affirmative response (score = 1)** |
| --- | --- | --- |
| Food depletion | Food didn’t last; no money for more | Often true / Sometimes true |
| Unaffordable balanced meals | Couldn’t afford balanced meals | Often true / Sometimes true |
| Meal skipping | Cut meal size or skipped meals due to cost | Yes (any frequency) |
| Recurrent meal skipping | Frequency of meal skipping due to cost | Almost every month / Some months |
| Reduced intake | Ate less than felt necessary due to cost | Yes |
| Hunger without eating | Hungry but didn’t eat due to cost | Yes |

Items were adapted from the USDA six-item food security module (12-month recall). Responses were dichotomized, with affirmative responses coded as 1 and non-affirmative responses coded as 0, and summed to generate a total food insecurity score ranging from 0 to 6 (higher scores indicate greater food insecurity). For the two statement items, “often true” and “sometimes true” were coded as affirmative; “never true” was coded as non-affirmative. For the meal skipping item, any affirmative frequency was coded as affirmative, whereas recurrent meal skipping was defined as occurring in “some months” or “almost every month.” Responses not ascertained or not provided were treated as missing.

**Supplementary Table 2. Assessment of depressive symptoms and depression: items, wording, and scoring criteria**

| **CES-D item** | **Scoring contribution** |
| --- | --- |
| **Depressive symptoms (CES-D-8)** |  |
| Felt depressed | Yes = 1, No = 0 |
| Everything was an effort | Yes = 1, No = 0 |
| Restless sleep | Yes = 1, No = 0 |
| Could not get going | Yes = 1, No = 0 |
| Felt lonely | Yes = 1, No = 0 |
| Felt sad | Yes = 1, No = 0 |
| Was happy | Yes = 0, No = 1 |
| Enjoyed life | Yes = 0, No = 1 |
| **Depression (binary outcome)** |  |
| Elevated depressive symptoms (Defined from CES-D-8 total score (range: 0–8) | In the main analyses, depression was defined as **CES-D-8 ≥ 3** (yes/no) |

Depressive symptoms were assessed using the 8-item Center for Epidemiologic Studies Depression scale (CES-D-8), capturing whether each symptom was experienced “much of the time” during the week prior to interview. Items were scored as 1 for “yes” and 0 for “no,” with the two positive-affect items (“was happy” and “enjoyed life”) reverse-coded (yes = 0; no = 1). Item scores were summed to generate a total CES-D-8 score ranging from 0 to 8, with higher scores indicating greater depressive symptom burden. For the primary analyses, depression was operationalized as elevated depressive symptoms defined by a CES-D-8 score ≥3 (binary yes/no).

**Supplementary Table 3. Definitions and characteristics of epigenetic aging measures**

| **Clock** | **Model** | **Features** | **Scale / unit** |
| --- | --- | --- | --- |
| Horvath (2013)^1^ | Multi-tissue age predictor trained across 51 tissues/cell types | 353 CpGs | Years |
| Hannum (2013)^2^ | Whole-blood age predictor | 71 CpGs | Years |
| DNAm PhenoAge (Levine, 2018)^3^ | DNAm predictor of “phenotypic age” derived from clinical biomarker composite and age | 513 CpGs | Years |
| Skin & Blood clock (Horvath, 2018)^4^ | Developed to better measure age in fibroblasts/skin-related tissues; applicable across multiple tissues | 391 CpGs | Years |
| Lin (2016)^5^ | Blood-based DNAm model trained on life expectancy | 99 CpGs | Years |
| Weidner (2014)^6^ | Minimal blood clock using selected age-related CpGs | 3 CpGs | Years |
| Vidal-Bralo (2016)^7^ | Adult-focused DNAm age predictor to improve calibration in adults | 8 CpGs | Years |
| DNAm GrimAge (Lu, 2019)^8^ | Composite built from DNAm surrogates of plasma proteins + DNAm smoking pack-years, then linked to time-to-death | DNAm surrogates (7 plasma proteins) + DNAm smoking pack-years | Years |
| Yang (2016)^9^ | Mitotic-like clock derived from PCGT promoter CpGs (epiTOC framework) | 385 CpGs | Score |
| Zhang (2017)^10^ | Mortality-associated DNAm signature selected from EWAS replication | 10 CpGs | Score |
| Bocklandt (2011)^11^ | Predictor derived in saliva; age-related methylation signal with a 2-CpG predictor | 2 CpGs | Score |
| Garagnani (2012)^12^ | Age-correlated methylation in ELOVL2 (validated in blood) | 1 CpG | Score |
| DunedinPoAm (Belsky, 2020)^13^ | Elastic-net DNAm algorithm trained on 18-biomarker “Pace of Aging” slopes across ages 26/32/38 | - | Years per chronological year |
| This table summarizes the DNA methylation–based epigenetic clocks included in the study. Clocks are grouped by their original training target and reported in their native scale. For clocks expressed in years, higher values indicate older DNAm-estimated age. Score-based measures (e.g., Yang and Zhang) reflect the original risk- or mitotic-related constructs and are not directly interpretable in units of years. DNAm GrimAge is a composite derived from DNAm-based surrogates of seven plasma proteins and DNAm smoking pack-years and is reported in years. DunedinPoAm is a rate measure expressed as years of physiological decline per one year of chronological age. References are listed in the order shown (1–13).  **Reference:**  1. Horvath S. DNA methylation age of human tissues and cell types. *Genome Biol*. 2013;14(10):R115. doi:10.1186/gb-2013-14-10-r115  2. Hannum G, Guinney J, Zhao L, et al. Genome-wide methylation profiles reveal quantitative views of human aging rates. *Mol Cell*. Jan 24 2013;49(2):359-367. doi:10.1016/j.molcel.2012.10.016  3. Levine ME, Lu AT, Quach A, et al. An epigenetic biomarker of aging for lifespan and healthspan. *Aging (Albany NY)*. Apr 18 2018;10(4):573-591. doi:10.18632/aging.101414  4. Horvath S, Oshima J, Martin GM, et al. Epigenetic clock for skin and blood cells applied to Hutchinson Gilford Progeria Syndrome and ex vivo studies. *Aging (Albany NY)*. Jul 26 2018;10(7):1758-1775. doi:10.18632/aging.101508  5. Lin Q, Weidner CI, Costa IG, et al. DNA methylation levels at individual age-associated CpG sites can be indicative for life expectancy. *Aging (Albany NY)*. Feb 2016;8(2):394-401. doi:10.18632/aging.100908  6. Weidner CI, Lin Q, Koch CM, et al. Aging of blood can be tracked by DNA methylation changes at just three CpG sites. *Genome Biol*. Feb 3 2014;15(2):R24. doi:10.1186/gb-2014-15-2-r24  7. Vidal-Bralo L, Lopez-Golan Y, Gonzalez A. Simplified Assay for Epigenetic Age Estimation in Whole Blood of Adults. *Front Genet*. 2016;7:126. doi:10.3389/fgene.2016.00126  8. Lu AT, Quach A, Wilson JG, et al. DNA methylation GrimAge strongly predicts lifespan and healthspan. *Aging (Albany NY)*. Jan 21 2019;11(2):303-327. doi:10.18632/aging.101684  9. Yang Z, Wong A, Kuh D, et al. Correlation of an epigenetic mitotic clock with cancer risk. *Genome Biol*. Oct 3 2016;17(1):205. doi:10.1186/s13059-016-1064-3  10. Zhang Y, Wilson R, Heiss J, et al. DNA methylation signatures in peripheral blood strongly predict all-cause mortality. *Nat Commun*. Mar 17 2017;8:14617. doi:10.1038/ncomms14617  11. Bocklandt S, Lin W, Sehl ME, et al. Epigenetic predictor of age. *PLoS One*. 2011;6(6):e14821. doi:10.1371/journal.pone.0014821  12. Garagnani P, Bacalini MG, Pirazzini C, et al. Methylation of ELOVL2 gene as a new epigenetic marker of age. *Aging Cell*. Dec 2012;11(6):1132-4. doi:10.1111/acel.12005  13. Belsky DW, Caspi A, Arseneault L, et al. Quantification of the pace of biological aging in humans through a blood test, the DunedinPoAm DNA methylation algorithm. *Elife*. May 5 2020;9doi:10.7554/eLife.54870 | | | |

**Supplementary Table 4. Baseline characteristics according to incident depression status**

| **Variables** | **Total (n=5547)** | **No incident depression (n=4227)** | **Incident depression (n=1320)** | ***P* value** |
| --- | --- | --- | --- | --- |
| **Age at baseline, years** | 66.51 ± 10.28 | 66.61 ± 10.32 | 66.21 ± 10.16 | 0.21 |
| **BMI, kg/m**2 | 28.47 ± 5.83 | 28.26 ± 5.64 | 29.13 ± 6.37 | **<0.001** |
| **Food insecurity score** | 0.58 ± 1.42 | 0.46 ± 1.27 | 0.94 ± 1.77 | **<0.001** |
| **Sex** |  |  |  | **<0.001** |
| Male | 2341(42.20) | 1902(45.00) | 439(33.26) |  |
| Female | 3206(57.80) | 2325(55.00) | 881(66.74) |  |
| **Race** |  |  |  | **<0.01** |
| White | 3981(71.77) | 3083(72.94) | 898(68.03) |  |
| Black | 856(15.43) | 639(15.12) | 217(16.44) |  |
| Hispanic | 526( 9.48) | 379( 8.97) | 147(11.14) |  |
| Other | 184( 3.32) | 126( 2.98) | 58( 4.39) |  |
| **Education** |  |  |  | **<0.001** |
| Below high school | 740(13.34) | 512(12.11) | 228(17.27) |  |
| High school | 1792(32.31) | 1342(31.75) | 450(34.09) |  |
| College or above | 3015(54.35) | 2373(56.14) | 642(48.64) |  |
| **Marital status** |  |  |  | **<0.001** |
| Married or partnered | 3775(68.05) | 2940(69.55) | 835(63.26) |  |
| Separated/Divorced/Widowed | 1558(28.09) | 1145(27.09) | 413(31.29) |  |
| Never married | 214( 3.86) | 142( 3.36) | 72( 5.45) |  |
| **Smoking status** |  |  |  | **0.02** |
| Never smokers | 2605(46.96) | 2022(47.84) | 583(44.17) |  |
| Ever smokers | 2942(53.04) | 2205(52.16) | 737(55.83) |  |
| **Drinking status** |  |  |  | **<0.01** |
| Never drinkers | 2376(42.83) | 1769(41.85) | 607(45.98) |  |
| Ever drinkers | 3171(57.17) | 2458(58.15) | 713(54.02) |  |
| **Physical activity** |  |  |  | **<0.001** |
| Inactive | 1455(26.23) | 1040(24.60) | 415(31.44) |  |
| Moderate | 1866(33.64) | 1384(32.74) | 482(36.52) |  |
| Vigorous | 2226(40.13) | 1803(42.65) | 423(32.05) |  |
| **Residence** |  |  |  | 0.50 |
| Urban | 2869(51.72) | 2205(52.16) | 664(50.30) |  |
| Suburban | 1207(21.76) | 911(21.55) | 296(22.42) |  |
| Rural | 1471(26.52) | 1111(26.28) | 360(27.27) |  |
| **Household income** |  |  |  | **<0.001** |
| Low | 1849(33.33) | 1307(30.92) | 542(41.06) |  |
| Moderate | 1847(33.30) | 1402(33.17) | 445(33.71) |  |
| High | 1851(33.37) | 1518(35.91) | 333(25.23) |  |
| **Food insecurity** |  |  |  | **<0.001** |
| High food security | 4789(86.33) | 3762(89.00) | 1027(77.80) |  |
| Low food security | 468( 8.44) | 301( 7.12) | 167(12.65) |  |
| Very low food security | 290( 5.23) | 164( 3.88) | 126( 9.55) |  |
| **Food depletion** | 863(15.56) | 545(12.89) | 318(24.09) | **<0.001** |
| **Unaffordable balanced meals** | 849(15.31) | 524(12.40) | 325(24.62) | **<0.001** |
| **Meal skipping** | 490( 8.83) | 299( 7.07) | 191(14.47) | **<0.001** |
| **Recurrent meal skipping** | 375( 6.76) | 223( 5.28) | 152(11.52) | **<0.001** |
| **Reduced intake** | 410( 7.39) | 247( 5.84) | 163(12.35) | **<0.001** |
| **Hunger without eating** | 213( 3.84) | 119( 2.82) | 94( 7.12) | **<0.001** |
| **Hypertension** | 3206(57.80) | 2359(55.81) | 847(64.17) | **<0.001** |
| **Diabetes** | 1176(21.20) | 864(20.44) | 312(23.64) | **0.01** |
| **Heart disease** | 1234(22.25) | 923(21.84) | 311(23.56) | 0.20 |
| **Stroke** | 401( 7.23) | 293( 6.93) | 108( 8.18) | 0.14 |
| **Cancer** | 880(15.86) | 669(15.83) | 211(15.98) | 0.93 |

Continuous variables are presented as mean ± standard deviation (SD), and categorical variables are presented as number (percentage [%]). P values are from two-sided tests comparing participants with and without incident depression (t test for continuous variables, and χ² test for categorical variables, as appropriate). Two-sided *P* values are presented without adjustment for multiple comparisons, with values <0.001 reported as <0.001. Incident depression was defined as CES-D-8 ≥3 during follow-up (Wave 13–16). Food insecurity was assessed at baseline (Wave 12) using the USDA 6-item scale and categorized as high food security (score 0–1), low food security (score 2–4), and very low food security (score 5–6).

**Supplementary Table 5. Baseline characteristics of the full analytic sample and the epigenetic subsample**

| **Variables** | **Full sample (n=5547)** | **Subsample**  **(n=1430)** |
| --- | --- | --- |
| **Age at baseline, years** | 66.51 ± 10.28 | 66.17 ± 9.48 |
| **BMI, kg/m**2 | 28.47 ± 5.83 | 28.60 ± 5.84 |
| **Food insecurity score** | 0.58 ± 1.42 | 0.56 ± 1.37 |
| **Sex** |  |  |
| Male | 2341(42.20) | 607(42.45) |
| Female | 3206(57.80) | 823(57.55) |
| **Race** |  |  |
| White | 3981(71.77) | 1048(73.29) |
| Black | 856(15.43) | 200(13.99) |
| Hispanic | 526( 9.48) | 150(10.49) |
| Other | 184( 3.32) | 32( 2.24) |
| **Education** |  |  |
| Below high school | 740(13.34) | 210(14.69) |
| High school | 1792(32.31) | 458(32.03) |
| College or above | 3015(54.35) | 762(53.29) |
| **Marital status** |  |  |
| Married or partnered | 3775(68.05) | 990(69.82) |
| Separated/Divorced/Widowed | 1558(28.09) | 361(25.46) |
| Never married | 214( 3.86) | 67( 4.72) |
| **Smoking status** |  |  |
| Never smokers | 2605(46.96) | 642(45.40) |
| Ever smokers | 2942(53.04) | 772(54.60) |
| **Drinking status** |  |  |
| Never drinkers | 2376(42.83) | 563(39.68) |
| Ever drinkers | 3171(57.17) | 856(60.32) |
| **Physical activity** |  |  |
| Inactive | 1455(26.23) | 350(24.72) |
| Moderate | 1866(33.64) | 469(33.12) |
| Vigorous | 2226(40.13) | 597(42.16) |
| **Residence** |  |  |
| Urban | 2869(51.72) | 750(52.82) |
| Suburban | 1207(21.76) | 309(21.76) |
| Rural | 1471(26.52) | 361(25.42) |
| **Household income** |  |  |
| Low | 1849(33.33) | 474(33.38) |
| Moderate | 1847(33.30) | 473(33.31) |
| High | 1851(33.37) | 473(33.31) |
| **Hypertension** | 3206(57.80) | 820(57.75) |
| **Diabetes** | 1176(21.20) | 316(22.25) |
| **Heart disease** | 1234(22.25) | 323(22.75) |
| **Stroke** | 401( 7.23) | 81( 6.40) |
| **Cancer** | 880(15.86) | 207(16.36) |
| **Incident depression during follow-up** | 1320(23.80) | 359(25.10) |

Continuous variables are presented as mean ± standard deviation (SD), and categorical variables are presented as number (percentage [%]). The epigenetic subsample consisted of participants with available DNA methylation data for epigenetic clock estimation. Incident depression during follow-up was defined as CES-D-8 ≥3 (Wave 13–16). Food insecurity was assessed at baseline (Wave 12) using the USDA 6-item scale and summarized as a total score (range: 0–6; higher scores indicate greater food insecurity).

**Supplementary Table 6. Distributions of epigenetic clocks and measures of epigenetic age acceleration**

| **Clock** | **Generation** | **Mean** | **Standard Deviation** | **Minimum** | **Maximum** |
| --- | --- | --- | --- | --- | --- |
| **Epigenetic Clocks** |  |  |  |  |  |
| Horvath 1 | 1 | 66.68 | 9.69 | 31.98 | 114.52 |
| Hannum | 1 | 55.18 | 9.19 | 32.01 | 89.66 |
| Levine | 2 | 57.87 | 9.94 | 26.72 | 100.20 |
| Horvath 2 | 1 | 70.32 | 8.83 | 41.40 | 99.97 |
| Lin | 1 | 59.18 | 11.18 | 7.44 | 133.27 |
| Weidner | 1 | 67.51 | 11.78 | 34.88 | 148.87 |
| VidalBralo | 1 | 64.06 | 6.19 | 44.40 | 109.95 |
| Yang | 1 | 0.07 | 0.02 | 0.04 | 0.23 |
| Zhang | 2 | -1.10 | 0.45 | -2.23 | 0.31 |
| Bocklandt | 1 | -0.39 | 0.08 | -0.86 | -0.13 |
| Garagnani | 1 | 0.72 | 0.07 | 0.45 | 0.99 |
| GrimAge | 2 | 68.29 | 8.49 | 46.01 | 90.24 |
| DunedinPoAm | 3 | 1.07 | 0.09 | 0.74 | 1.38 |
| **Epigenetic Aging Acceleration** |  |  |  |  |  |
| Horvath 1 | 1 | 0.00 | 6.45 | -34.53 | 48.03 |
| Hannum | 1 | 0.00 | 5.24 | -30.71 | 34.67 |
| Levine | 2 | 0.00 | 6.86 | -27.93 | 42.19 |
| Horvath 2 | 1 | 0.00 | 4.40 | -24.59 | 18.13 |
| Lin | 1 | 0.00 | 7.71 | -54.95 | 57.21 |
| Weidner | 1 | 0.00 | 10.83 | -30.54 | 71.66 |
| VidalBralo | 1 | 0.00 | 5.03 | -20.89 | 40.26 |
| Yang | 1 | 0.00 | 0.02 | -0.03 | 0.15 |
| Zhang | 2 | 0.00 | 0.43 | -1.22 | 1.40 |
| Bocklandt | 1 | 0.00 | 0.07 | -0.44 | 0.23 |
| Garagnani | 1 | 0.00 | 0.05 | -0.25 | 0.25 |
| GrimAge | 2 | 0.00 | 4.54 | -11.74 | 22.89 |
| DunedinPoAm | 3 | 1.07 | 0.09 | 0.74 | 1.38 |

Values are shown for the epigenetic subsample with available DNA methylation data. Epigenetic clocks are reported on their native scale; for clocks expressed in years, higher values indicate older DNAm-estimated age. Score-based measures (e.g., Yang, Zhang, Bocklandt and Garagnani) are reported in their original units and are not directly interpretable in years. Epigenetic age acceleration (AgeAccel) was calculated as the residual from regressing each epigenetic clock (or score) on chronological age (age at Wave 13), with positive residuals indicating accelerated epigenetic aging. DunedinPoAm is a pace-of-aging measure (years of physiological decline per one year of chronological age) and is therefore presented on its original scale rather than as an AgeAccel residual. Generation indicates the conceptual generation of each measure (first-generation chronological age clocks vs second-generation risk- and phenotype-related clocks).

**Supplementary Table 7. Associations of food insecurity and its individual components with incident depression**

|  | **Independent association** | | **Mutually adjusted association** | |  |
| --- | --- | --- | --- | --- | --- |
|  | **HR (95% CI)** | ***P* value** | **HR (95% CI)** | ***P* value** | **PAF** |
| **Food depletion** | 1.65(1.42,1.90) | <0.001 | 1.25(1.01,1.56) | 0.044 | 9.2% |
| **Unaffordable balanced meals** | 1.72(1.49,1.99) | <0.001 | 1.43(1.16,1.77) | <0.001 | 9.9% |
| **Meal skipping** | 1.55(1.31,1.84) | <0.001 | 0.94(0.65,1.36) | 0.729 | 4.6% |
| **Recurrent meal skipping** | 1.58(1.32,1.90) | <0.001 | 1.06(0.73,1.52) | 0.772 | 3.8% |
| **Reduced intake** | 1.57(1.31,1.87) | <0.001 | 1.00(0.74,1.34) | 0.977 | 4.0% |
| **Hunger without eating** | 1.71(1.37,2.14) | <0.001 | 1.20(0.88,1.62) | 0.244 | 2.7% |
| **Food insecurity** |  |  |  |  | 8.9% |
| High food security | 1(reference) |  |  |  | - |
| Low food security | 1.54(1.29,1.85) | <0.001 |  |  | 4.5% |
| Very low food security | 1.90(1.55,2.33) | <0.001 |  |  | 4.5% |

Hazard ratios (HRs) and 95% confidence intervals (CIs) were estimated using multivariable Cox proportional hazards models. “Independent association” models evaluated each food insecurity component separately. “Mutually adjusted association” models included all six USDA food insecurity components simultaneously to estimate their independent contributions. Models were adjusted for age at baseline, body mass index, sex, race, education, marital status, smoking status, drinking status, physical activity, residence, and household income. High food security served as the reference category for categorical food security status. Population attributable fractions (PAFs) represent the proportion (%) of incident depression cases attributable to the exposure under the model assumptions. Two-sided unadjusted *P* values are shown, with values <0.001 reported as <0.001.

**Supplementary Table 8. E-values for the association between food insecurity severity and incident depression**

|  | **HR (95% CI)** | ***P* value** | **E value (L-bound)** |
| --- | --- | --- | --- |
| **Food depletion** | 1.65(1.42,1.90) | <0.001 | 2.69 (2.19) |
| **Unaffordable balanced meals** | 1.72(1.49,1.99) | <0.001 | 2.83 (2.34) |
| **Meal skipping** | 1.55(1.31,1.84) | <0.001 | 2.47 (1.95) |
| **Recurrent meal skipping** | 1.58(1.32,1.90) | <0.001 | 2.54 (1.97) |
| **Reduced intake** | 1.57(1.31,1.87) | <0.001 | 2.52 (1.95) |
| **Hunger without eating** | 1.71(1.37,2.14) | <0.001 | 2.81 (2.08) |
| **Food insecurity** |  |  |  |
| High food security | 1(reference) |  | - |
| Low food security | 1.58(1.32,1.88) | <0.001 | 2.54 (1.97) |
| Very low food security | 1.94(1.58,2.37) | <0.001 | 3.29 (2.54) |

Hazard ratios (HRs) and 95% confidence intervals (CIs) were estimated using multivariable Cox proportional hazards models adjusted for age at baseline, body mass index, sex, race, education, marital status, smoking status, drinking status, physical activity, residence, and household income. High food security served as the reference category for categorical food security status. E-values quantify the minimum strength of association (on the risk ratio scale) that an unmeasured confounder would need to have with both the exposure and incident depression, conditional on the measured covariates, to fully explain away the observed association; the L-bound corresponds to the E-value for the lower limit of the 95% CI. Two-sided unadjusted P values are shown, with values <0.001 reported as <0.001.

**Supplementary Table 9. Associations between food insecurity and epigenetic age acceleration**

| **Epigenetic clock** | **Beta (95% CI)** | ***P* value** |
| --- | --- | --- |
| Horvath AgeAccel | 0.007 (-0.036, 0.050) | 0.747 |
| Hannum AgeAccel | 0.022 (-0.020, 0.065) | 0.300 |
| Skin&Blood AgeAccel | 0.025 (-0.018, 0.068) | 0.260 |
| Lin AgeAccel | -0.008 (-0.052, 0.036) | 0.718 |
| Weidner AgeAccel | 0.015 (-0.028, 0.059) | 0.497 |
| Vidal-Bralo AgeAccel | 0.003 (-0.040, 0.046) | 0.881 |
| Yang AgeAccel | 0.035 (-0.007, 0.077) | 0.104 |
| Bocklandt AgeAccel | **-0.060 (-0.102, -0.017)** | **0.006** |
| Garagnani AgeAccel | 0.008 (-0.036, 0.051) | 0.729 |
| PhenoAgeAccel | 0.004 (-0.039, 0.047) | 0.861 |
| Zhang AgeAccel | **0.042 (0.001, 0.083)** | **0.045** |
| GrimAgeAccel | 0.003 (-0.034, 0.040) | 0.869 |
| DunedinPoAm | 0.003 (-0.038, 0.044) | 0.882 |

β coefficients and 95% confidence intervals (CIs) were estimated using multivariable linear regression models in the epigenetic subsample, modelling baseline food insecurity score as a continuous exposure (per 1-point increment). Epigenetic age acceleration was defined as the residual from regressing each DNAm age estimate on chronological age (age at Wave 13), with positive residuals indicating accelerated epigenetic aging. DunedinPoAm was analysed on its original scale (years of physiological decline per one year of chronological age). Models were adjusted for age at baseline, sex, race, education, marital status, smoking status, drinking status, physical activity, residence, household income, and body mass index. Two-sided unadjusted P values are shown, with values <0.001 reported as <0.001. N denotes participants with non-missing values for the corresponding epigenetic measure.

**Supplementary Table 10. Associations between epigenetic age acceleration and incident depression**

| **Epigenetic clock** | **Linear association between epigenetic age acceleration and incident depression** | | **Associations between tertiles of epigenetic age acceleration and incident depression** | | | |
| --- | --- | --- | --- | --- | --- | --- |
|  | Per 1-SD increase | | **Middle tertile** | | **Highest tertile** | |
|  | HR (95% CI) | *P* value | HR (95% CI) | *P* value | HR (95% CI) | *P* value |
| Horvath AgeAccel | 1.01 (0.91, 1.12) | 0.905 | 0.93 (0.71, 1.20) | 0.565 | 1.01 (0.78, 1.30) | 0.958 |
| Hannum AgeAccel | 1.09 (0.98, 1.20) | 0.123 | 1.29 (0.99, 1.69) | 0.064 | 1.28 (0.98, 1.69) | 0.074 |
| Skin&Blood AgeAccel | 1.11 (0.99, 1.23) | 0.066 | 1.23 (0.94, 1.61) | 0.129 | 1.27 (0.97, 1.66) | 0.080 |
| Lin AgeAccel | 1.04 (0.94, 1.16) | 0.435 | 1.05 (0.81, 1.37) | 0.718 | 1.21 (0.93, 1.56) | 0.158 |
| Weidner AgeAccel | 1.11 (1.00, 1.24) | 0.043 | 1.06 (0.82, 1.39) | 0.641 | 1.18 (0.91, 1.53) | 0.221 |
| Vidal-Bralo AgeAccel | 1.13 (1.02, 1.25) | 0.024 | 1.20 (0.92, 1.56) | 0.183 | 1.28 (0.98, 1.67) | 0.067 |
| Yang AgeAccel | 1.03 (0.93, 1.13) | 0.611 | 1.11 (0.85, 1.46) | 0.448 | 1.08 (0.82, 1.43) | 0.563 |
| Bocklandt AgeAccel | 1.02 (0.92, 1.13) | 0.743 | 1.13 (0.87, 1.46) | 0.355 | 1.00 (0.76, 1.31) | 1.000 |
| Garagnani AgeAccel | 1.04 (0.94, 1.16) | 0.441 | 1.03 (0.79, 1.34) | 0.832 | 1.10 (0.84, 1.42) | 0.489 |
| PhenoAgeAccel | 1.12 (1.02, 1.24) | 0.021 | **1.37 (1.04, 1.81)** | **0.023** | **1.52 (1.16, 1.98)** | **0.002** |
| Zhang AgeAccel | 1.18 (1.06, 1.32) | 0.002 | 1.10 (0.83, 1.44) | 0.516 | 1.32 (1.01, 1.74) | 0.043 |
| GrimAgeAccel | 1.05 (0.93, 1.18) | 0.436 | 1.36 (1.04, 1.79) | 0.026 | 1.19 (0.88, 1.61) | 0.272 |
| DunedinPoAm | 1.02 (0.91, 1.14) | 0.737 | 1.08 (0.83, 1.40) | 0.581 | 1.03 (0.79, 1.35) | 0.818 |

Hazard ratios (HRs) and 95% confidence intervals (CIs) for incident depression were estimated using multivariable Cox proportional hazards models in the epigenetic subsample. Epigenetic age acceleration (AgeAccel) was defined as the residual from regressing each epigenetic clock on chronological age (age at Wave 13), with positive residuals indicating accelerated epigenetic aging. Linear associations are reported per 1–standard deviation (SD) increase in AgeAccel. For categorical analyses, AgeAccel was grouped into tertiles, with the lowest tertile as the reference category. Models were adjusted for age at baseline, sex, race, education, marital status, smoking status, drinking status, physical activity, residence, household income, and body mass index. Two-sided unadjusted P values are shown, with values <0.001 reported as <0.001. N denotes participants with non-missing values for the corresponding epigenetic measure.

**Supplementary Table 11. Mediation analyses evaluating accelerated epigenetic aging in the association between food insecurity and incident depression**

| Epigenetic clock | Total effect  HR (95% CI) | Natural direct effect HR (95% CI) | Natural indirect effect HR (95% CI) | Percentage mediated % (95% CI) |
| --- | --- | --- | --- | --- |
| Horvath AgeAccel | 1.229 (1.167, 1.298) | 1.229 (1.166, 1.298) | 1.000 (0.997, 1.003) | 0.003 (−1.295, 1.323) |
| Hannum AgeAccel | 1.230 (1.165, 1.303) | 1.229 (1.165, 1.302) | 1.001 (0.998, 1.005) | 0.311 (−1.080, 2.328) |
| Skin & Blood AgeAccel | 1.229 (1.163, 1.306) | 1.227 (1.162, 1.305) | 1.002 (0.999, 1.007) | 0.808 (−0.733, 3.771) |
| Lin AgeAccel | 1.230 (1.163, 1.297) | 1.231 (1.163, 1.299) | 1.000 (0.996, 1.003) | −0.148 (−1.939, 1.351) |
| Weidner AgeAccel | 1.232 (1.159, 1.307) | 1.232 (1.160, 1.306) | 1.000 (0.996, 1.003) | −0.148 (−1.950, 1.350) |
| Vidal-Bralo AgeAccel | 1.232 (1.163, 1.300) | 1.233 (1.164, 1.301) | 0.999 (0.994, 1.002) | −0.531 (−2.794, 1.027) |
| Yang AgeAccel | 1.229 (1.159, 1.299) | 1.223 (1.153, 1.296) | 1.005 (0.997, 1.013) | 2.383 (−1.274, 6.815) |
| Bocklandt AgeAccel | 1.230 (1.160, 1.297) | 1.230 (1.161, 1.297) | 1.000 (0.993, 1.008) | 0.229 (−3.925, 3.950) |
| Garagnani AgeAccel | 1.229 (1.166, 1.299) | 1.229 (1.166, 1.299) | 1.000 (0.997, 1.003) | 0.016 (−1.298, 1.464) |
| PhenoAgeAccel | 1.229 (1.165, 1.296) | 1.226 (1.162, 1.292) | 1.003 (0.998, 1.009) | 1.433 (−0.976, 4.482) |
| **Zhang AgeAccel** | **1.230 (1.163, 1.304)** | **1.217 (1.148, 1.292)** | **1.011 (1.003, 1.022)** | **5.284 (1.489, 11.682)** |
| GrimAgeAccel | 1.227 (1.163, 1.299) | 1.222 (1.155, 1.296) | 1.005 (0.998, 1.013) | 2.214 (−0.689, 6.937) |
| DunedinPoAm | 1.229 (1.162, 1.299) | 1.225 (1.155, 1.293) | 1.003 (0.998, 1.011) | 1.670 (−0.975, 5.960) |

Mediation analyses decomposed the association between baseline food insecurity score (modelled continuously per 1-point increment) and incident depression into the total effect (TE), natural direct effect (NDE) and natural indirect effect (NIE) through accelerated epigenetic aging. Epigenetic age acceleration (AgeAccel) was defined as the residual from regressing each epigenetic clock on chronological age (age at Wave 13), with positive residuals indicating accelerated epigenetic aging; DunedinPoAm was analysed on its original scale. TE, NDE and NIE are reported as hazard ratios (HRs) with 95% confidence intervals (CIs). The percentage mediated was calculated on the HR scale as (NIE-1)/(TE-1)*100% and 95% CIs were obtained by bootstrap resampling. Models were adjusted for age at baseline, sex, race, education, marital status, smoking status, drinking status, physical activity, residence, household income, and body mass index. N denotes participants with non-missing values for the corresponding epigenetic measure. Two-sided unadjusted P values are presented elsewhere, with values <0.001 reported as <0.001.

**Supplementary Table 12. Sensitivity analyses for the association between food insecurity and incident depression**

|  | **Cases** | **Total** | **HR (95% CI)** | ***P* value** |
| --- | --- | --- | --- | --- |
| **Sensitivity analysis 1. Results based on unimputed data** | | | | |
| Food depletion | 318 | 863 | 1.63(1.41,1.89) | <0.001 |
| Unaffordable balanced meals | 325 | 849 | 1.7(1.46,1.97) | <0.001 |
| Meal skipping | 191 | 490 | 1.51(1.27,1.79) | <0.001 |
| Recurrent meal skipping | 152 | 375 | 1.52(1.26,1.83) | <0.001 |
| Reduced intake | 163 | 410 | 1.57(1.31,1.88) | <0.001 |
| Hunger without eating | 94 | 213 | 1.66(1.32,2.08) | <0.001 |
| **Food insecurity** |  |  |  |  |
| High food security | 1,027 | 4,789 | 1(reference) |  |
| Low food security | 167 | 468 | 1.54(1.29,1.85) | <0.001 |
| Very low food security | 126 | 290 | 1.88(1.53,2.32) | <0.001 |
| P for trend |  |  |  | <0.001 |
| **Food insecurity score** | - | - | 1.13(1.09,1.17) | <0.001 |
| **Sensitivity analysis 2. Further adjustment for major chronic diseases** | | | | |
| Food depletion | 318 | 863 | 1.61(1.39,1.87) | <0.001 |
| Unaffordable balanced meals | 325 | 849 | 1.69(1.46,1.95) | <0.001 |
| Meal skipping | 191 | 490 | 1.53(1.29,1.81) | <0.001 |
| Recurrent meal skipping | 152 | 375 | 1.56(1.29,1.87) | <0.001 |
| Reduced intake | 163 | 410 | 1.53(1.28,1.83) | <0.001 |
| Hunger without eating | 94 | 213 | 1.69(1.35,2.12) | <0.001 |
| **Food insecurity** |  |  |  |  |
| High food security | 1,027 | 4,789 | 1(reference) |  |
| Low food security | 167 | 468 | 1.54(1.29,1.85) | <0.001 |
| Very low food security | 126 | 290 | 1.90(1.55,2.33) | <0.001 |
| P for trend |  |  |  | <0.001 |
| **Food insecurity score** | - | - | 1.13(1.09,1.17) | <0.001 |
| **Sensitivity analysis 3. Exclusion of participants with baseline major chronic diseases** | | | | |
| Food depletion | 71 | 229 | 2.02(1.49,2.72) | <0.001 |
| Unaffordable balanced meals | 75 | 228 | 2.27(1.67,3.10) | <0.001 |
| Meal skipping | 45 | 139 | 1.85(1.30,2.62) | <0.001 |
| Recurrent meal skipping | 35 | 109 | 1.83(1.25,2.68) | 0.002 |
| Reduced intake | 40 | 123 | 1.9(1.31,2.73) | <0.001 |
| Hunger without eating | 25 | 69 | 2.15(1.38,3.36) | <0.001 |
| **Food insecurity** |  |  |  |  |
| High food security | 247 | 1,363 | 1(reference) |  |
| Low food security | 36 | 111 | 2.11(1.44,3.09) | <0.001 |
| Very low food security | 32 | 94 | 2.36(1.57,3.54) | <0.001 |
| P for trend |  |  |  | <0.001 |
| **Food insecurity score** | - | - | 1.18(1.11,1.27) | <0.001 |
| **Sensitivity analysis 4. Exclusion of early events within the first two years of follow-up** | | | | |
| Food depletion | 288 | 833 | 2.02(1.49,2.72) | <0.001 |
| Unaffordable balanced meals | 295 | 819 | 2.27(1.67,3.10) | <0.001 |
| Meal skipping | 173 | 472 | 1.85(1.30,2.62) | <0.001 |
| Recurrent meal skipping | 138 | 361 | 1.83(1.25,2.68) | 0.002 |
| Reduced intake | 144 | 391 | 1.9(1.31,2.73) | <0.001 |
| Hunger without eating | 88 | 207 | 2.15(1.38,3.36) | <0.001 |
| **Food insecurity** |  |  |  |  |
| High food security | 921 | 4,683 | 1(reference) |  |
| Low food security | 150 | 451 | 1.62(1.34,1.95) | <0.001 |
| Very low food security | 113 | 277 | 1.99(1.60,2.46) | <0.001 |
| P for trend |  |  |  | <0.001 |
| **Food insecurity score** | - | - | 1.14(1.10,1.18) | <0.001 |
| **Sensitivity analysis 5. Five-year landmark analysis** | | | | |
| Food depletion | 97 | 642 | 2.02(1.49,2.72) | <0.001 |
| Unaffordable balanced meals | 102 | 626 | 2.27(1.67,3.10) | <0.001 |
| Meal skipping | 65 | 364 | 1.85(1.30,2.62) | <0.001 |
| Recurrent meal skipping | 53 | 276 | 1.83(1.25,2.68) | 0.002 |
| Reduced intake | 55 | 302 | 1.9(1.31,2.73) | <0.001 |
| Hunger without eating | 32 | 151 | 2.15(1.38,3.36) | <0.001 |
| **Food insecurity** |  |  |  |  |
| High food security | 402 | 4,164 | 1(reference) |  |
| Low food security | 51 | 352 | 1.46(1.07,2.00) | 0.02 |
| Very low food security | 41 | 205 | 1.93(1.36,2.75) | <0.001 |
| P for trend |  |  |  | <0.001 |
| **Food insecurity score** | - | - | 1.13(1.07,1.20) | <0.001 |
| **Sensitivity analysis 6. Propensity score–matched analysis** | | | | |
| Food depletion | 318 | 863 | 1.65(1.42,1.90) | <0.001 |
| Unaffordable balanced meals | 325 | 849 | 1.72(1.49,1.99) | <0.001 |
| Meal skipping | 191 | 490 | 1.55(1.31,1.84) | <0.001 |
| Recurrent meal skipping | 152 | 375 | 1.58(1.32,1.90) | <0.001 |
| Reduced intake | 163 | 410 | 1.57(1.31,1.87) | <0.001 |
| Hunger without eating | 94 | 213 | 1.71(1.37,2.14) | <0.001 |
| **Food insecurity** |  |  |  |  |
| High food security | 1,027 | 4,789 | 1(reference) |  |
| Low food security | 167 | 468 | 1.58(1.32,1.88) | <0.001 |
| Very low food security | 126 | 290 | 1.94(1.58,2.37) | <0.001 |
| P for trend |  |  |  | <0.001 |
| **Food insecurity score** | - | - | 1.13(1.09,1.17) | <0.001 |

Multivariable Cox proportional hazards models were used to estimate hazard ratios (HRs) and 95% confidence intervals (CIs) for the associations between baseline food insecurity and incident depression. Robustness was evaluated across six prespecified sensitivity scenarios: (1) analyses using the unimputed dataset, (2) further adjustment for major chronic diseases, (3) exclusion of participants with baseline major chronic diseases, (4) exclusion of early events occurring within the first 2 years of follow-up, (5) a 5-year landmark analysis conditioned on being event-free at 5 years, and (6) propensity score–matched analyses. P for trend was obtained by modelling food security status as an ordinal variable. Food insecurity was assessed using the USDA 6-item scale and analysed as both a categorical exposure (high food security [reference], low food security and very low food security) and a continuous score (per 1-point increment).

**
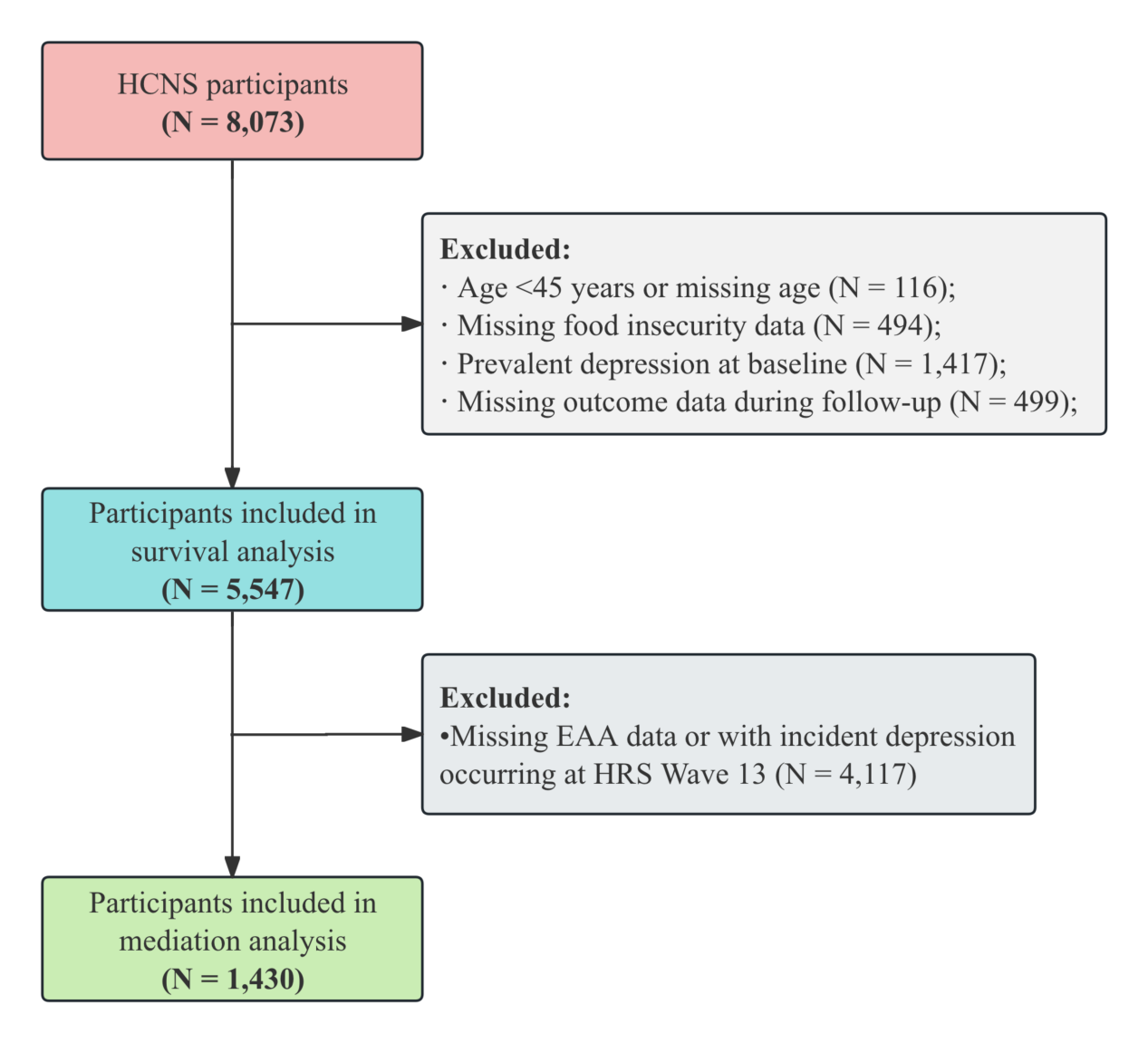
**

**Supplementary Figure 1. Flowchart of participant selection**

Of 8,073 respondents in the 2013 Health Care and Nutrition Study (HCNS), we excluded participants aged <45 years or with missing age (n = 116), missing food insecurity data (n = 494), prevalent depression at baseline (n = 1,417), or missing depression outcome data during follow-up (n = 499), leaving 5,547 participants for the primary survival analyses. For analyses incorporating epigenetic measures, we further restricted to participants with available DNA methylation–derived epigenetic age acceleration (EAA) measures in Health and Retirement Study (HRS) wave 13 (n = 1,430). HCNS, Health Care and Nutrition Study; HRS, Health and Retirement Study; EAA, epigenetic age acceleration.

**
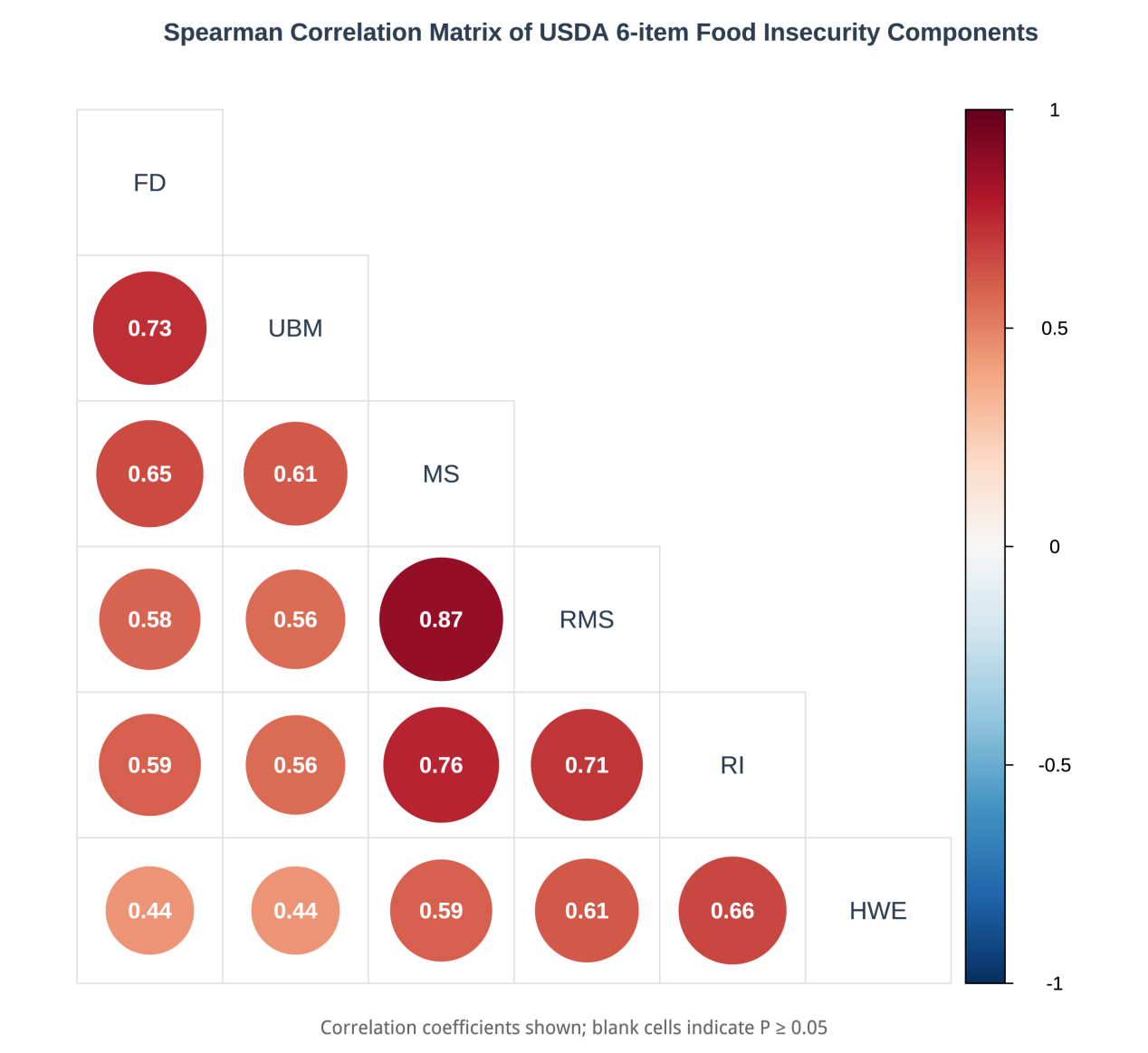
**

**Supplementary Figure 2. Spearman correlations among items of the USDA six-item food insecurity scale**

Abbreviations: FD, food depletion; UBM, unaffordable balanced meals; MS, meal skipping; RMS, recurrent meal skipping; RI, reduced intake; HWE, hunger without eating.

**
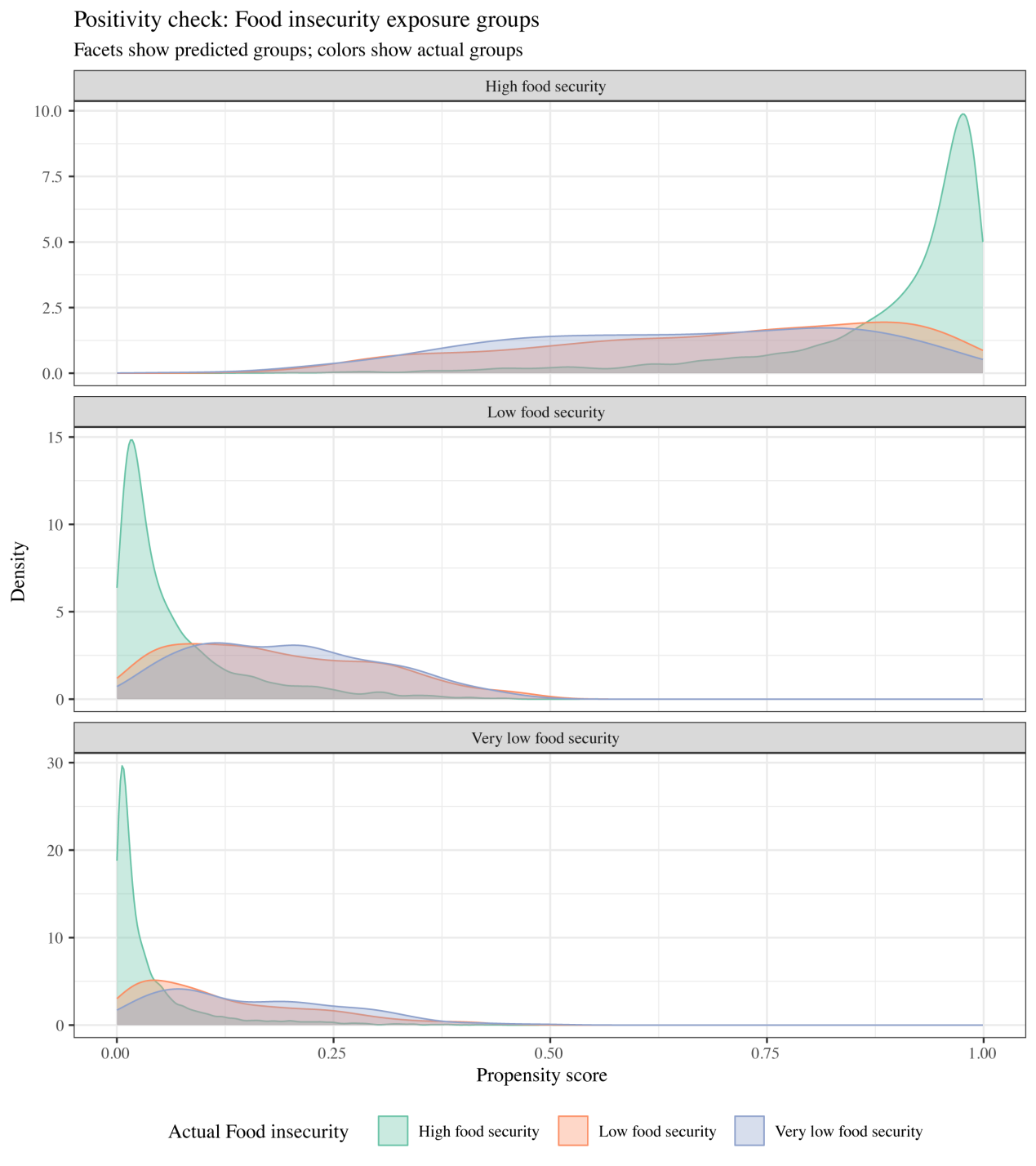
**

**Supplementary Figure 3. Propensity score distributions across food insecurity categories to assess the positivity assumption**

Kernel density plots of the estimated propensity score for each food security group (high, low, and very low). Panels correspond to the predicted exposure group, whereas coloured curves denote the observed food security category. Adequate overlap of propensity score distributions across groups supports the positivity assumption for propensity score–based analyses.

**
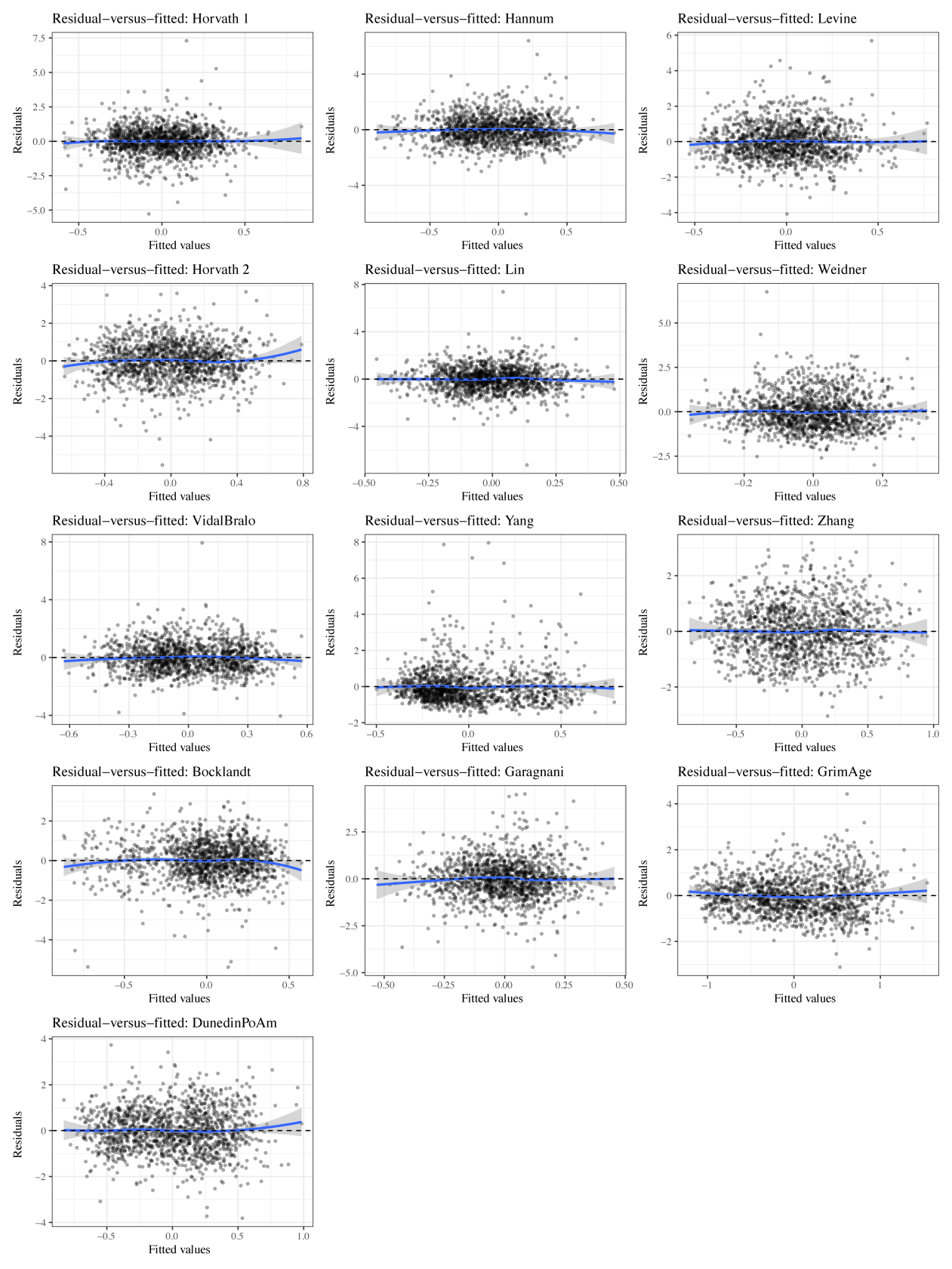
**

**Supplementary Figure 4. Residual-versus-fitted plots for models of epigenetic aging measures**

Residual–versus–fitted plots for the linear models used to derive epigenetic age acceleration measures. Each panel shows residuals against fitted values from regressing the corresponding DNAm clock on chronological age (Wave 13); the dashed line denotes zero residuals and the smooth curve indicates the fitted trend.

**
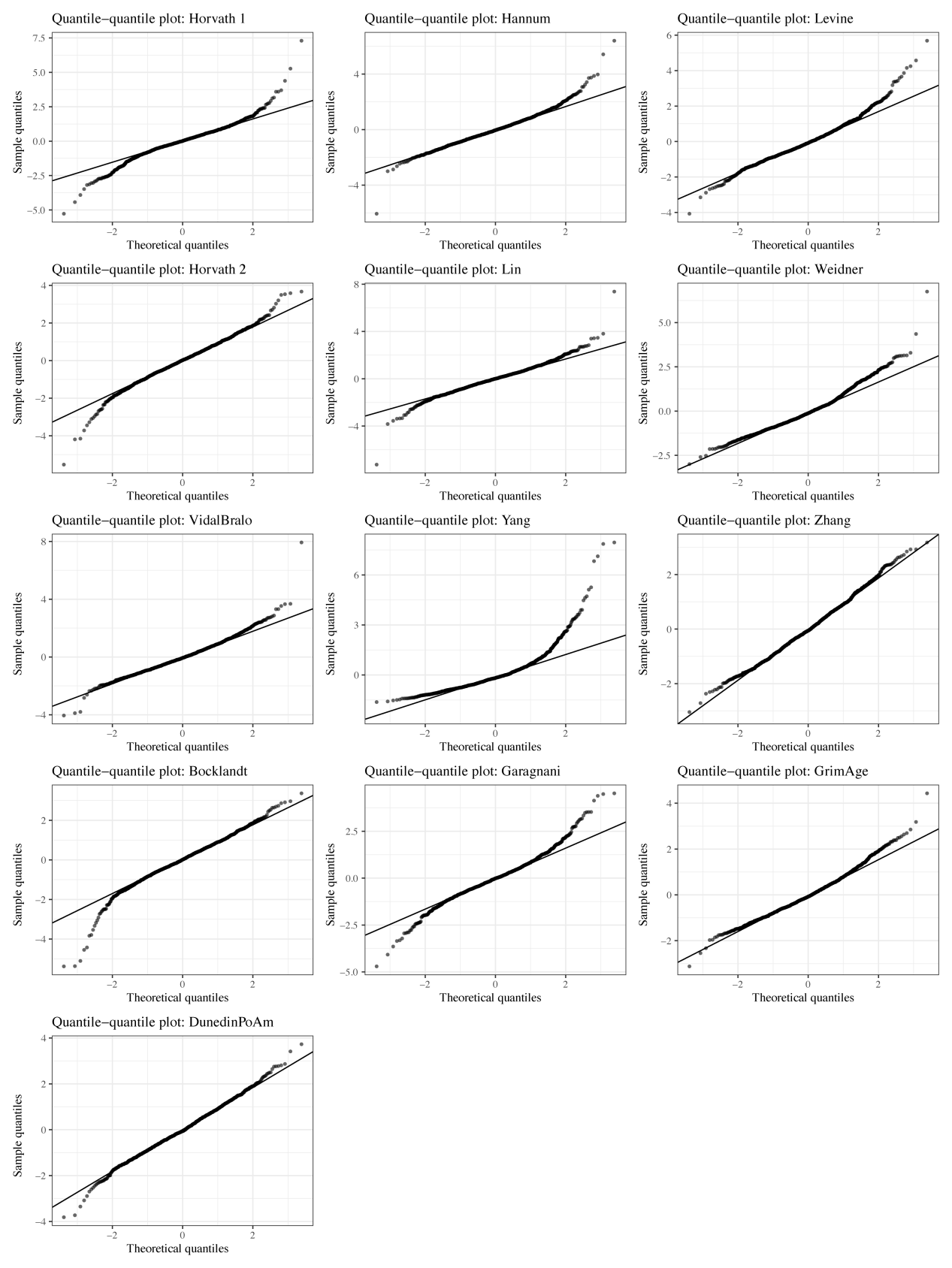
**

**Supplementary Figure 5. Quantile–quantile plots for models of epigenetic aging measures**

Q–Q plots of residuals from the age-acceleration models for each DNAm clock, compared with the theoretical normal distribution. Points closely tracking the reference line indicate approximate normality; departures at the tails reflect residual non-normality.

**
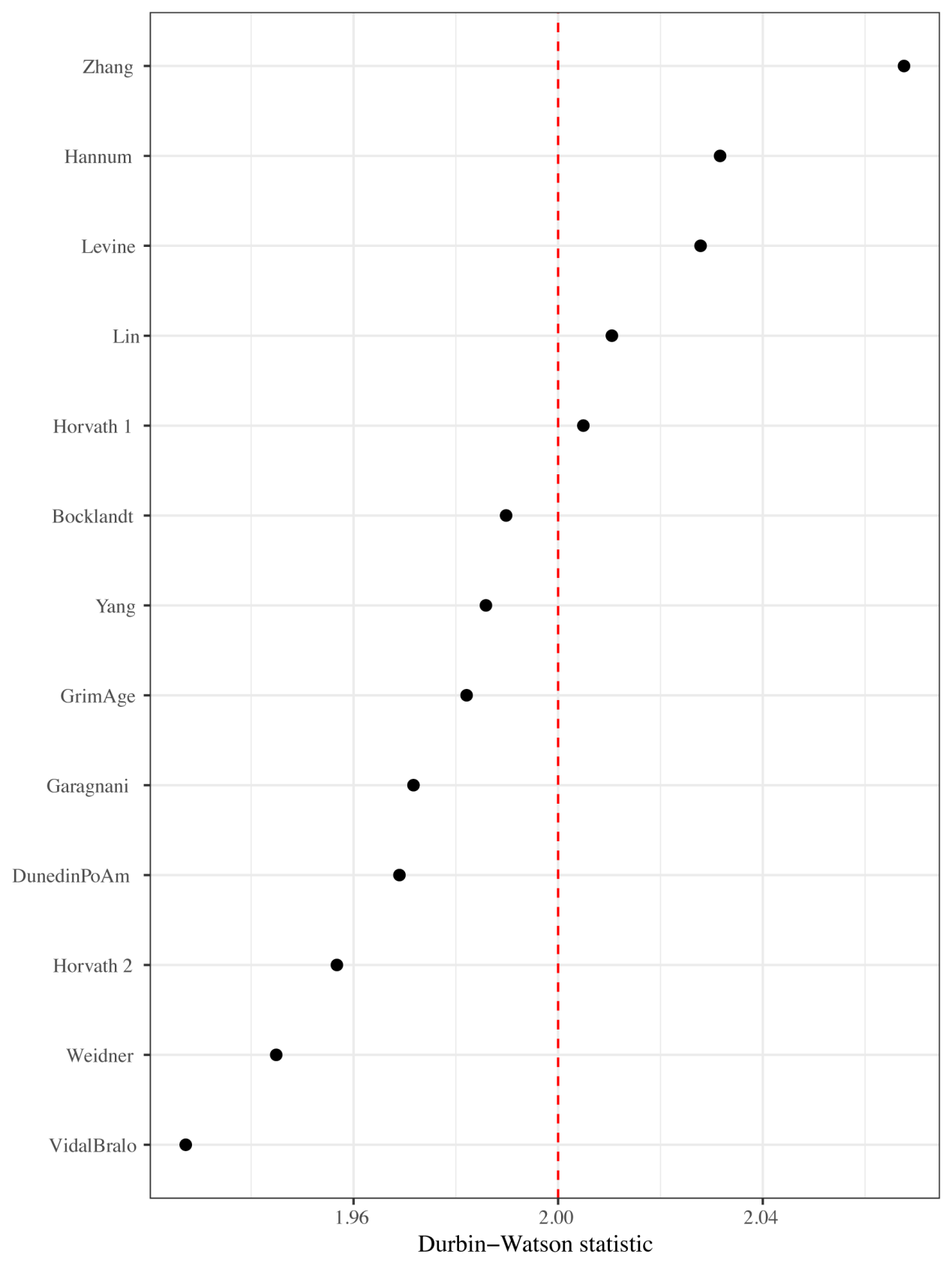
**

**Supplementary Figure 6. Durbin–Watson statistics for models of epigenetic aging measures**

Durbin–Watson statistics are shown for the regression models used to compute epigenetic age acceleration for each DNAm clock. Values near 2 (dashed line) indicate that model residuals are approximately independent.

**
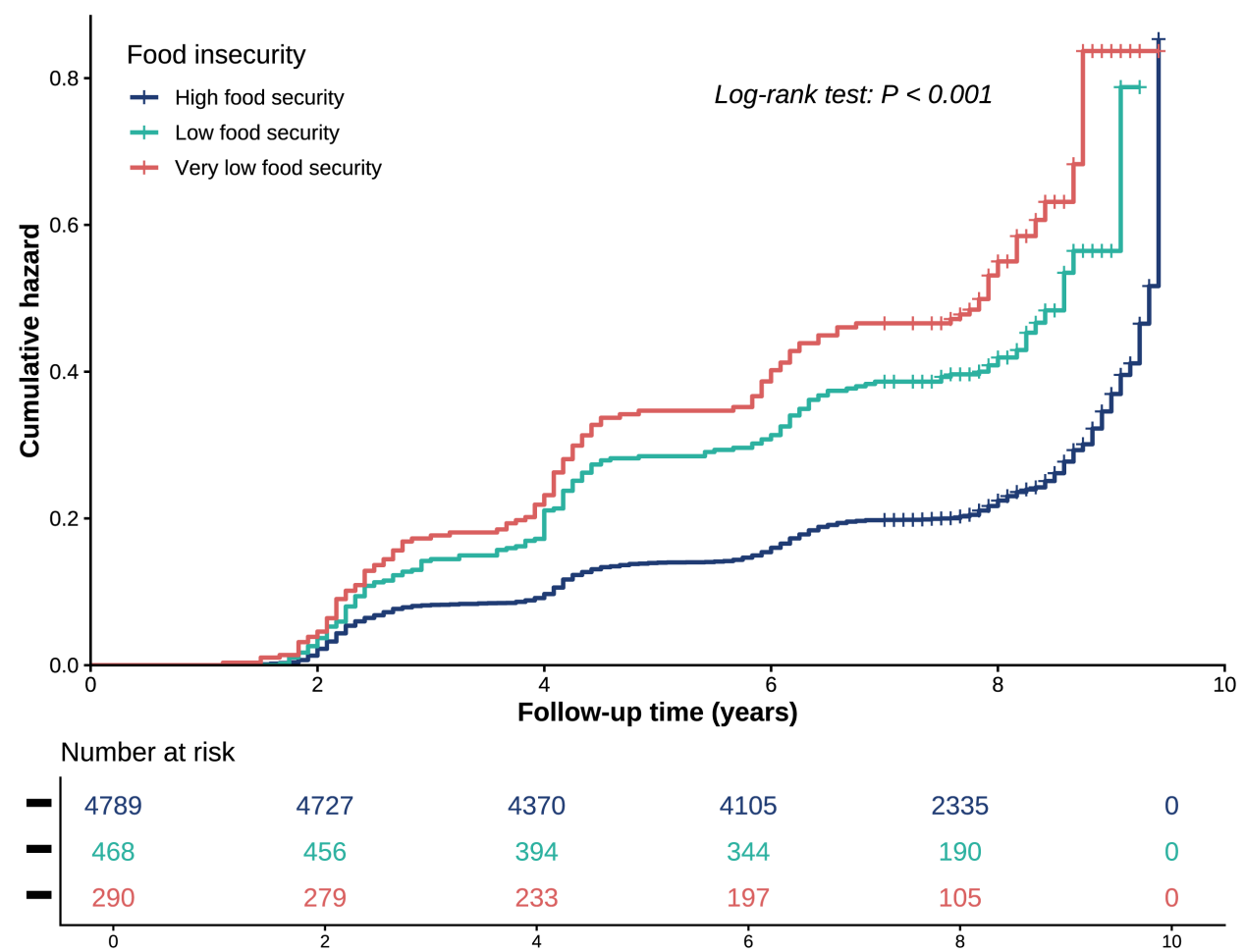
**

**Supplementary Figure 7. Kaplan–Meier curves for incident depression by baseline food security status**

Kaplan–Meier estimates of time to incident depression are shown for high food security (USDA 6-item score 0–1), low food security (score 2–4), and very low food security (score 5–6). Tick marks denote censoring. Numbers at risk are provided below the plot. Group differences were assessed using the log-rank test (two-sided).

**
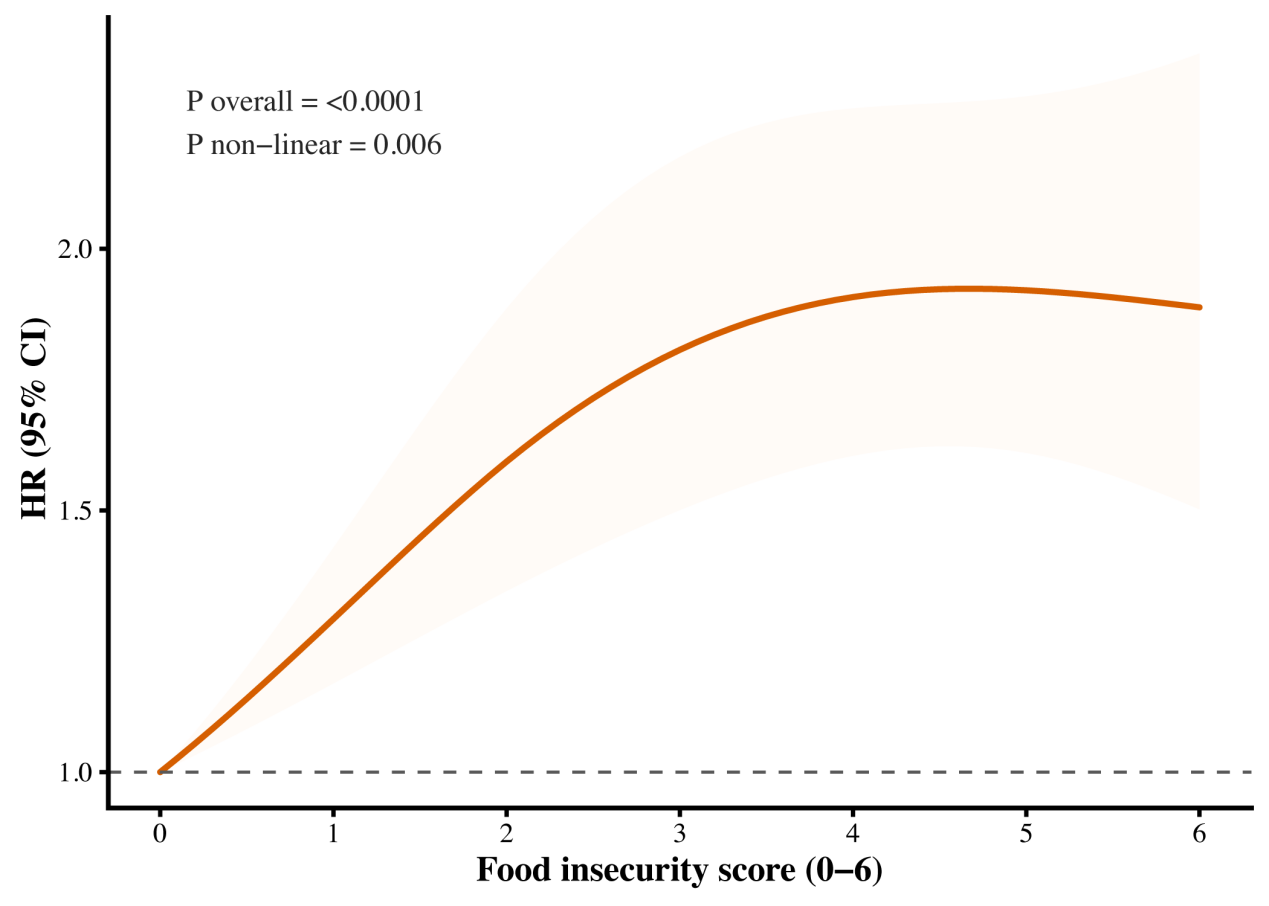
**

**Supplementary Figure 8. Dose–response association between food insecurity score and incident depression**
Hazard ratios (HRs) and 95% confidence intervals (CIs) for incident depression are shown across the USDA six-item food insecurity score (range 0–6), modelled using restricted cubic splines in multivariable Cox proportional hazards models. The solid line denotes the adjusted HR and the shaded area the 95% CI; the dashed horizontal line indicates HR = 1. High food security (score 0) served as the reference. Models were adjusted for age at baseline, sex, race, education, marital status, smoking status, drinking status, physical activity, residence, household income, and body mass index . P values are shown for the overall association and for non-linearity.

**
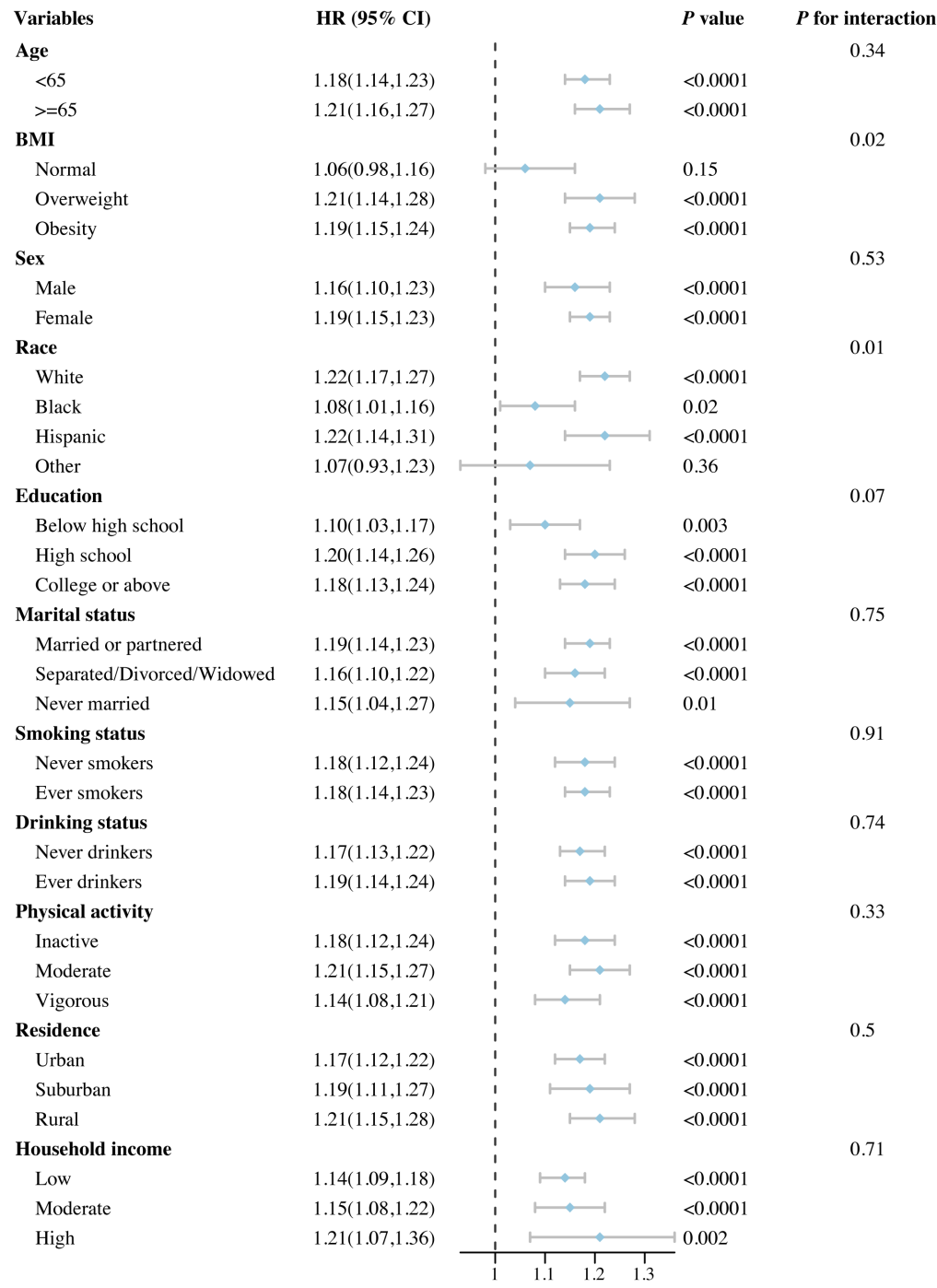
**

**Supplementary Figure 9. Stratified associations between food insecurity score and incident depression across demographic and clinical subgroups**
Forest plots show hazard ratios (HRs) and 95% confidence intervals (CIs) for incident depression per 1-point increase in the USDA six-item food insecurity score, estimated using multivariable Cox models within prespecified subgroups. Models were adjusted for age at baseline, sex, race, education, marital status, smoking status, drinking status, physical activity, residence, household income, and body mass index, except for the stratifying variable. *P* for interaction was derived from models including a multiplicative interaction term between the food insecurity score and each subgroup variable. Two-sided P values are shown (<0.0001 reported as <0.0001). Age group and BMI group were treated as categorical variables.
